# Gallic Acid Alleviates Gut Dysfunction and Boosts Immune and Antioxidant Activities in Puppies Under Environmental Stress Based on Microbiome–Metabolomics Analysis

**DOI:** 10.3389/fimmu.2021.813890

**Published:** 2022-01-14

**Authors:** Kang Yang, Xiaolin Deng, Shiyan Jian, Meiyu Zhang, Chaoyu Wen, Zhongquan Xin, Limeng Zhang, Aorigeile Tong, Shibin Ye, Pinfeng Liao, Zaili Xiao, Shansong He, Fan Zhang, Jinping Deng, Lingna Zhang, Baichuan Deng

**Affiliations:** ^1^ Guangdong Laboratory for Lingnan Modern Agriculture, Guangdong Provincial Key Laboratory of Animal Nutrition Control, National Engineering Research Center for Breeding Swine Industry, College of Animal Science, South China Agricultural University, Guangzhou, China; ^2^ Department of Urology, Ganzhou People’s Hospital, Ganzhou, China; ^3^ College of Animal Science and Technology, Guangdong Polytechnic of Science and Trade, Guangzhou, China; ^4^ Research Center of Pet Nutrition, Guangzhou Qingke Biotechnology Co., Ltd., Guangzhou, China

**Keywords:** environmental stress, gallic acid, puppy, antioxidant, inflammatory response, microbiome, metabolomics

## Abstract

Early-life exposure to environmental stress disrupts the gut barrier and leads to inflammatory responses and changes in gut microbiota composition. Gallic acid (GA), a natural plant polyphenol, has received significant interest for its antioxidant, anti-inflammatory, and antimicrobial properties that support the maintenance of intestinal health. To assess whether dietary supplementation of GA alleviates environmental stress, a total of 19 puppies were randomly allocated to the following three dietary treatments for 2 weeks: 1) basal diet (control (CON)); 2) basal diet + transportation (TS); and 3) basal diet with the addition of 500 mg/kg of GA + transportation (TS+GA). After a 1-week supplementation period, puppies in the TS and TS+GA groups were transported from a stressful environment to another livable location, and puppies in the CON group were then left in the stressful environment. Results indicated that GA markedly reduced the diarrhea rate in puppies throughout the trial period and caused a moderate decline of serum cortisol and HSP-70 levels after transportation. Also, GA alleviated the oxidative stress and inflammatory response caused by multiple environmental stressors. Meanwhile, puppies fed GA had a higher abundance of fecal Firmicutes and *Lactobacillus* and lower Proteobacteria, *Escherichia–Shigella*, and *Clostridium_sensu_stricto_1* after transportation. As a result, the TS+GA group had the highest total short-chain fatty acids and acetic acid. Also, the fecal and serum metabolomics analyses revealed that GA markedly reversed the abnormalities of amino acid metabolism, lipid metabolism, carbohydrate metabolism, and nucleotide metabolism caused by stresses. Finally, Spearman’s correlation analysis was carried out to explore the comprehensive microbiota and metabolite relationships. Overall, dietary supplementation of GA alleviates oxidative stress and inflammatory response in stressed puppies by causing beneficial shifts on gut microbiota and metabolites that may support gut and host health.

## Introduction

Stress response is a ubiquitous physiological response elicited when the threat to the homeostasis is perceived by the organism due to environmental, physical, or psychological stimuli (1). Early-life exposure to a specific environment can influence the development and function of multiple organs and systems, including the central nervous, gastrointestinal, and immune systems (2–4). Current evidence suggests that the hypothalamic–pituitary–adrenal (HPA) axis is the major pathway that controls the production of the stress hormones, glucocorticoids (GC) in response to various environmental factors (e.g., oxidative stress, heat, and osmotic stress). A series of metabolic and immune-suppressive effects (5) are elicited by GC, acting through the glucocorticoid receptor. Specifically, the HPA axis is activated by the secretion of corticotropin-releasing hormone (CRH) from the hypothalamus, which induces the anterior pituitary gland to release adrenocorticotropic hormone (ACTH), and then ACTH stimulates the adrenal cortex to release the GC, mainly cortisol (COR), which negatively regulates CRH production to terminate the stress response cascade (6–8). Moreover, heat shock proteins (HSPs), a kind of stress-induced proteins ubiquitously found in germs and mammals (9–11), are heavily involved in dealing with environmental stress (12). Particularly, HSP-70 serves as a molecular chaperone to protect cells against the stresses of various types and origins. A recent study has demonstrated that HSP-70 helps to maintain and stabilize the intestinal tight junctions, as a result generating a stronger intestinal barrier in the ileum of stressed animals (13, 14). Simultaneously, environmental stressors trigger the production of intracellular reactive oxygen species (ROS) that can disrupt the cellular antioxidant defense system (15). Stress-induced production of ROS may be mediated by the inflammatory response because inflammation is associated with high levels of ROS, and strong stressors can induce an inflammatory response (16).

Stress not only affects the physiological and stress system but also destroys gut microbiota (GM) (17–19). The human body is inhabited by trillions of microorganisms that participate in nutrient metabolism and influence the health and immune responses of the host (20–22). *Lactobacillus* and *Bifidobacterium* are the main genera of probiotic bacteria, which enhance the host immune system and favorably modulate gastrointestinal physiology (23, 24). Moreover, the producers of short-chain fatty acids (SCFAs), the phylum Firmicutes and the genera *Faecalibacterium* and *Roseburia*, may also be considered beneficial bacteria (25–28) because SCFAs are a carbon energy source for intestinal epithelial cells and can induce the development of intestinal Treg cell with potent anti-inflammatory functions (29–31). Conversely, the pathogenic bacteria Enterobacteriaceae (belong to the phylum Proteobacteria), a family including *Escherichia*, *Shigella*, *Proteus*, and *Klebsiella*, is often associated with the development of systemic inflammation (32, 33). It is increasingly recognized that the acute and chronic stressors that activate the HPA axis can modulate GM and may be one causal factor in gut dysbiosis (1). In support, recent evidence has begun to connect GM and its metabolites to gastrointestinal diseases, inflammation, and psychological metrics in humans suffering from multiple stressors (8, 17, 18). Collectively, these studies provide preliminary evidence that GM may respond to environmental stress.

Polyphenol performs antioxidant and anti-inflammatory properties and can modulate oxidative stress and inflammatory signaling (34–36). Growing evidence indicates that polyphenol contributes to gut health *via* the modulation of colon microbiota composition (37–39). Gallic acid (GA), also known as 3,4,5-trihydroxybenzoic acid, is a natural polyphenol compound present in fruits, vegetables, and herbal medicines (40). It has been reported that GA effectively inhibited inflammation (41, 42) and oxidation (43, 44) *in vitro* and *in vivo* and altered metabolic and bacterial profiles in the colitis model (45). As far as we know, there is little discussion about whether GA can relieve the damage caused by multiple stressors. Based on previous research, we hypothesize that multiple stressors can cause inflammation and oxidative stress by promoting the growth of pathogenic bacteria species, thereby causing diarrhea; and dietary supplementation of GA may have a role in alleviating these symptoms.

Beagle dogs are considered excellent models for human microbiome research because of the high similarities in structures and functions between dog and human microbiomes (46). To determine whether changing environment and adding GA are efficacious in preventing the deleterious effects of stress on antioxidative and immune system activity, we transported puppies from a stressful environment to a livable environment. In detail, we evaluated the diarrhea rate, physiological stress, antioxidant capacity, inflammatory response, and metabolites by dietary supplementation of GA at 500 mg/kg before and after transportation. In parallel, the 16S rRNA gene sequencing was adopted to monitor microbiota alterations, and untargeted metabolomics based on ultra-performance liquid chromatography–Orbitrap–tandem mass spectrometry (UPLC-Orbitrap-MS/MS) analysis method was employed to capture changes in different metabolic pathways and potential metabolic biomarkers.

## Materials and Methods

### Animals and Diet

All experimental procedures were authorized by the Experimental Animal Ethics Committee of South China Agricultural University (Approval number: 2019188) and were performed following the guidelines of the Laboratory Animal Center at the South China Agricultural University. Animal welfare was monitored by research and animal care staff daily.

A total of 19 beagle dogs (**Table 1**) were selected in this study and were housed individually in pens (1.35 × 0.70 × 0.75 m kennels) under an indoor relative humidity and temperature of 96% ± 3% and 29°C ± 1°C, respectively (outdoor relative humidity and temperature were 99% ± 1% and 32°C ± 2°C, respectively) at a 12-h dark–light cycle at the National Canine Laboratory Animal Resource Bank, Guangzhou General Pharmaceutical Research Institute Co., Ltd (Guangzhou, China). All dogs were dewormed and vaccinated, and no drugs (such as antibiotics) that may alter the GM were given 1 month before the experiment. The blood samples were collected for serum biochemistry and blood routine examination 1 day before the trial. All blood routine and serum biochemistry data were within the normal range except for alkaline phosphatase, creatinine, creatine kinase, mean corpuscular hemoglobin, and lymph (**Table S1**), indicating that puppies under high temperature and high humidity remained in a stressed state.

**Table 1:** Detailed information of beagle dogs in this study.

Group	Sample size (male:female)	Age (month)	Initial body weight (kg)	BCS^1^
CON	6 (3:3)	3.56 ± 0.32	5.32 ± 0.91	5.42 ± 0.49
TS	6 (2:4)	3.62 ± 0.34	5.05 ± 0.52	5.17 ± 0.41
TS+GA	7 (3:4)	3.54 ± 0.30	5.23 ± 0.62	5.29 ± 0.49

CON, control; TS, transportation; GA, gallic acid.

^1^BCS, body condition score; all dogs were weighed, and BCS was assessed using a 9-point scale (47) before morning feeding. Data were expressed as mean ± SD.

Ground corn, flour, fish fat, chicken meal, beef powder, fish meal, soybean meal, amino acid, vitamin, and mineral premixes constituted the basal extruded diets. The chemical and energy composition of the basal diet is listed in **Table 2**. The basal diet meets all the nutrient recommendations by the Association of American Feed Control Officials (AAFCO, 2017) for puppies (48). Dogs were fed 100 g of diet twice daily (08:00 and 17:00) to meet the required energy needs based on the calculated metabolizable energy content of the basal diet according to the National Research Council (NRC, 2006) (49). They had free access to fresh water *ad libitum*. GA (purity > 99%) was purchased from Wufeng Chicheng Biotech Co., Ltd (Yichang, China). The dose of GA supplemented was based on previous studies (50) with minor modifications. After the adaptation period, 500 mg/kg of GA were mixed with the basal diet and individually dosed for each dog during the trial period. The daily dose of GA was divided and added equally to each of the two planned daily meals.

**Table 2:** The chemical and energy composition of basal diet tested.

Items^1^	Basal diet^2^
DM (%)	90.53
OM (% DM)	92.84
CP (% DM)	23.91
Acid-hydrolyzed fat (% DM)	4.56
TDF (% DM)	3.95
GE (kJ/g DM)	17.00

^1^DM, dry matter; OM, organic matter; CP, crude protein; TDF, total dietary fiber; GE, gross energy.

^2^Extruded diet: corn flour, flour, fish fat, chicken meal, beef powder, imported fish meal, soybean meal, calcium hydrophosphate, calcium chloride, lysine, methionine, vitamin A, vitamin D, vitamin E, copper sulfate, ferrous sulfate, zinc sulfate, and manganese sulfate.

### Experimental Design

**Figure 1:**
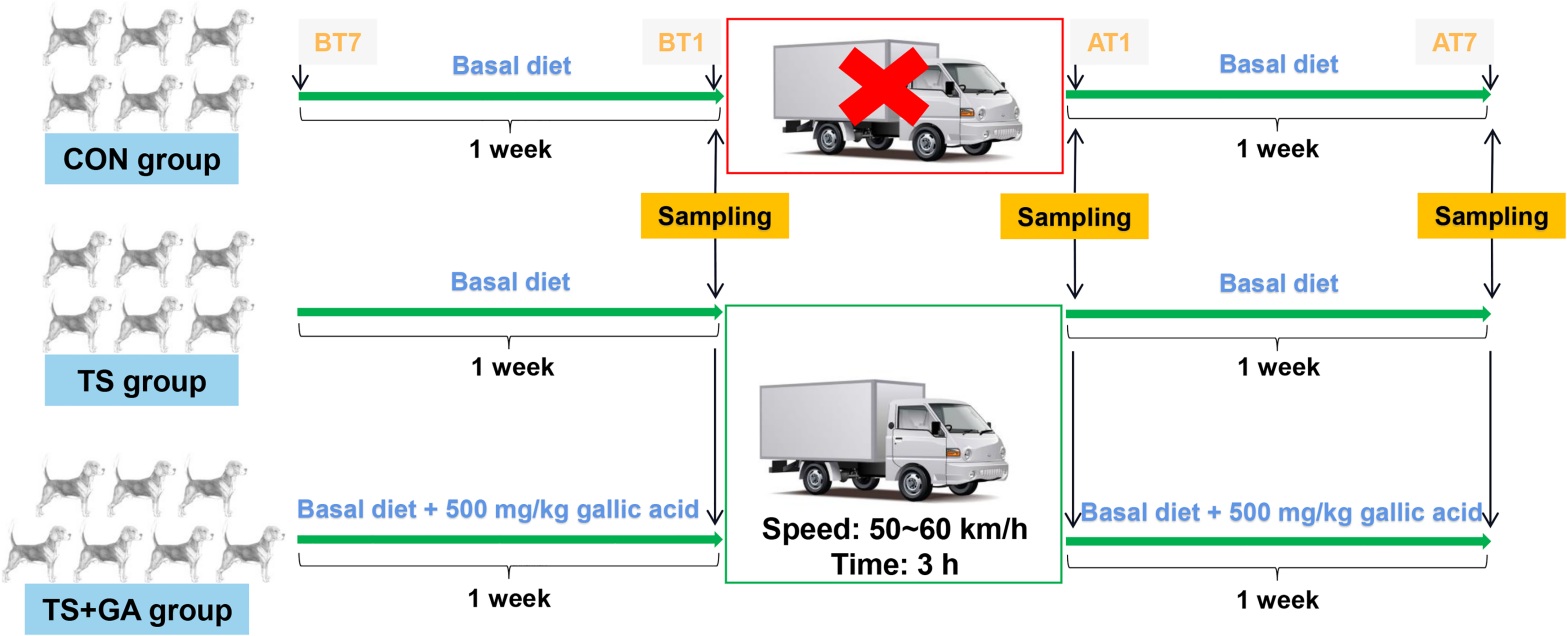
Schematic representation of the study design. BT7, the 7th day before transportation; BT1, the 1st day before transportation; AT1, the 1st day after transportation; AT7, the 7th day after transportation. The CON group was fed basal diet with no transportation (n = 6), the TS group was fed basal diet with transportation (n = 6), and the TS+GA group was fed basal diet+500 mg/kg of gallic acid (GA) with transportation (n = 7).

After 4 weeks of adaptation to a basal diet, these puppies were randomly allocated to one of the three dietary treatments: 1) basal diet (control group, CON group), 2) basal diet (transportation stress group, TS group), and 3) basal diet with the addition of 500 mg/kg of GA (TS+GA group). The experimental period was 14 days including 7 to 1 days before transportation (BT7–BT1) and 1 to 7 days after transportation (AT1–AT7). Puppies in the TS and TS+GA groups were exposed to the road transportation for 3 h (from 14:00 to 17:00) at a speed range of 50**~**60 km/h on day 7 of the experiment in a thermostatic truck at 26°C with 50% in humidity, and no environmental changes we made to the CON group during the study. Thirteen puppies in the TS and TS+GA groups were transported to the Laboratory Animal Center Building at the South China Agricultural University and housed individually in pens (1.2 × 1.0 × 1.1 m kennels) under a constant temperature and humidity (23°C and 70%, respectively) with a light/dark cycle of 12 h. All dogs were continued on their respective diets for another week and given access to toys for behavioral enrichment at all times and to exercise outside of their cages and socialize with each other or humans at least once a day. The study design is depicted in **Figure 1**.

### Chemical Analysis of Diet

Throughout the trial period, a 200-g basal diet was collected weekly and was kept in the refrigerator at −20°C. Feed samples were dried in the oven and were ground through a 1-mm screen for chemical composition analysis. The dry matter (DM) and organic matter (OM) were determined for the diets according to AOAC (2000; method 950.46 for water and method 942.05 for crude ash) (51). Acid-hydrolyzed fat was analyzed by a fatty analyzer (FT640, Guangzhou, Grand Analytical Instrument Co., Ltd) according to AOAC (2000; method 920.39 for ether extract) (51). The crude protein (CP) was done by using the Kjeldahl method with semi-automatic Kjeldahl apparatus (VAPODEST 200, C. Gerhardt GmbH & Co. KG, Germany) and following the Official Method of AOAC (2000; method 954.01 for crude protein) (51). The total dietary fiber (TDF) content was analyzed using an automatic fiber analyzer (FIBRETHERM FT12, C. Gerhardt GmbH & Co. KG, Germany) and AOAC (2000; method 962.09 for crude fiber) (51). Diet was analyzed for GE by oxygen bomb calorimeter (IKA C 200, IKA (Guangzhou) Instrument Equipment Co., Ltd, Guangzhou, China).

### Fresh Fecal Sample Collection and Analysis

During the whole experimental period of 2 weeks, fecal scores (FS) described by Middelbos et al. (52) were assessed every day. On BT1, AT1, and AT7, fresh fecal samples were collected from the pen floor of each dog within 15 min of defecation. An aliquot for SCFAs and branched-chain fatty acids (BCFAs) measurement was stored at −80°C until analysis. An aliquot of the feces was collected and transferred to a 5-ml sterile fecal collection tube (BIORISE) for microbiota measurement, snap-frozen on liquid N_2_, and stored at −80°C until DNA extraction. Finally, an aliquot for metabolomics analysis was snap-frozen on liquid N_2_ and stored at −80°C until analysis.

### Blood Sample Collection and Analysis

On BT1, AT1, and AT7 after overnight fasting, a 5-ml blood sample was collected from each dog by forelimb vein and left to stand for 30 min before centrifugation at 3,500*×g* at room temperature for 15 min. After centrifugation, the supernatants were aliquoted into microcentrifuge tubes and stored at −80°C for further analysis. Serum glutathione peroxidase (GSH-Px), malondialdehyde (MDA), total antioxidant capacity (T-AOC), and superoxide dismutase (SOD) were detected using commercial kits (Nanjing Jiancheng Bioengineering Institute, Nanjing, China) according to the manufacturer’s protocol. Serum COR, GC, ACTH, HSP-70, immunoglobulin G (IgG), tumor necrosis factor-alpha (TNF-α), interferon-γ (IFN-γ), and interleukin 4 (IL-4) were measured using commercial ELISA kits (MEIMIAN, Jiangsu Meimian Industrial Co., Ltd., Jiangsu, China). Finally, an aliquot for serum metabolomics analysis was snap-frozen on liquid N_2_ and stored at −80°C until analysis.

### 16S rRNA High-Throughput Sequencing

#### DNA Extraction, Amplification, and Sequencing

On BT1, AT1, and AT7, fresh fecal samples were collected from the pen floor of each dog within 15 min of defecation. Total genome DNA from fresh fecal samples was extracted using the cetyltrimethylammonium bromide method. DNA concentration and purity were monitored on 1% agarose gels. According to the concentration, DNA was diluted to 1 ng/µl using sterile water. 16S rRNA genes of 16S V3–V4 were amplified using the primers 341F (5′-CCTAYGGGRBGCASCAG-3′) and 806R (5′-GGACTACNNGGGTATCTAAT-3′) with the barcode. All PCRs were carried out with 15 µl of Phusion^®^ High-Fidelity PCR Master Mix (New England Biolabs) with 2 µM of forward and reverse primers and about 10 ng of template DNA. Thermal cycling consisted of initial denaturation at 98°C for 1 min, followed by 30 cycles of denaturation at 98°C for 10 s, annealing at 50°C for 30 s, and elongation at 72°C for 30 s, followed by 72°C for 5 min. The same volume of 1× loading buffer (contained SYB green) was mixed with PCR products (in equidensity ratios) and then operated with electrophoresis on 2% agarose gel for detection. Then, the mixture of PCR products was purified with Qiagen Gel Extraction Kit (Qiagen, Germany). Sequencing libraries were generated using the TruSeq^®^ DNA PCR-Free Sample Preparation Kit (Illumina, USA) following the manufacturer’s recommendations, and index codes were added. The library quality was assessed on the Qubit@ 2.0 Fluorometer (Thermo Scientific) and Agilent Bioanalyzer 2100 system. At last, the library was sequenced on an Illumina NovaSeq platform, and 250-bp paired-end reads were generated.

#### Bioinformatics Analysis

Paired-end reads were merged using FLASH (V1.2.7, http://ccb.jhu.edu/software/FLASH/) (53). Quality filtering on the raw tags was performed to obtain the high-quality clean tags (54) according to the QIIME (V1.9.1, http://qiime.org/scripts/split_libraries_fastq.html) (55) quality-controlled process. The tags were compared with the reference database (Silva database, https://www.arb-silva.de/) using the UCHIME algorithm (UCHIME, http://www.drive5.com/usearch/manual/uchime_algo.html) (56) to detect chimera sequences, and then the chimera sequences were removed (57). Then the effective tags are finally obtained.

Sequences analyses were performed by Uparse software (Uparse v7.0.1001, http://drive5.com/uparse/) (58). Sequences with ≥97% similarity were assigned to the same operational taxonomic units (OTUs). For each representative sequence, the Silva Database (http://www.arb-silva.de/) (59) was used based on the Mothur algorithm to annotate taxonomic information. Multiple sequence alignment was conducted using the MUSCLE software (Version 3.8.31, http://www.drive5.com/muscle/) (60) to study the phylogenetic relationship of different OTUs. Alpha diversity indices, including Observed_species, Chao1, Shannon, Simpson, ACE, and PD_whole_tree, were calculated with QIIME (Version 1.7.0) and displayed with R software (Version 2.15.3). Beta diversity on weighted UniFrac was calculated by QIIME software (Version 1.9.1). Principal coordinate analysis (PCoA) based on weighted UniFrac distances was displayed by WGCNA package, stat packages, and ggplot2 package in R software (Version 2.15.3). The linear discriminant analysis (LDA) effect size (LEfSe) was processed with the default setting of LDA score ≥4 using LEfSe software (http://huttenhower.sph.harvard.edu/lefse/). Correlation Network was performed using the OmicStudio tools at https://www.omicstudio.cn/tool. Function prediction of bacteria was conducted using PICRUSt (http://picrust.github.com/picrust/).

### Fecal Short-Chain Fatty Acid and Branched-Chain Fatty Acid Analyses

#### Sample Solution Preparation

The fresh fecal samples collected on BT1, AT1, and AT7 were pretreated, and extraction of SCFAs and BCFAs was performed as follows. The frozen stool samples were placed on ice to thaw, and a 0.2-g fecal sample was added with 1 ml of ultra-pure water. After vortex for 2 min, the samples were sonicated in an ice bath for 10 min and then centrifuged at 14,000 rpm for 10 min at 4°C. The supernatant was promptly transferred to a 2-ml centrifuge tube, and then a total of 20 μl of 25% metaphosphoric acid solution and 0.25-g anhydrous sodium sulfate were added to acidification and salting out, respectively. After vortex for 2 min, 1 ml of methyl *tert*-butyl ether was added, and the vortex was continued for 5 min, and the supernatant was further centrifuged at 14,000 rpm for another 10 min at 4°C to remove the precipitation. Finally, the upper extraction solution was harvested and filtered through 0.22-µm Millipore pore membrane filters to a 2-ml sample vial. Samples were stored at −20°C until gas chromatography–MS (GC-MS) analysis. All steps above were performed at 4°C or on ice.

#### Gas Chromatography–Mass Spectrometry Quantitative Analysis

The quantitative analysis of SCFAs and BCFAs was carried out using the GCMS-QP2020 system (Shimadzu, Tokyo, Japan). The gas chromatography was equipped with an auto-injector AOC-20i (Shimadzu) and coupled to a flame ionization detector. The chromatographic separation was performed on a DB-FFAP capillary column (30 m × 0.25 mm × 0.25 μm). Sample (0.6 μl) was injected with a 30:1 split ratio using an autosampler. The injection port was set to a temperature of 250°C. The initial temperature of the column was 80°C for 2 min and increased to 150°C at a rate of 10°C/min for 2 in, and to 180°C at a rate of 15°C/min for 5 min. The total run time was 18 min. Helium (He; 99.999%) was the carrier gas with a flow rate of 3 ml/min. The MS parameters were electron impact mode at ionization energy of 70 eV. The ion source and interface temperatures were 230°C and 250°C, respectively. The solvent delay time was 1 min, 230°C. The acquisition mode was selected at ion monitoring mode with a scan interval of 0.3 s.

### Fecal and Serum Untargeted Metabolomics Analyses

#### Sample Processing

The fresh fecal and serum samples collected on BT1, AT1, and AT7 were processed as described previously (61) with slight modifications. Briefly, frozen stool samples stored at −80°C were thawed at 4°C. Approximately 60 mg of sample was weighed and put into 2-ml round-bottom microcentrifuge tubes. Metabolites were extracted by adding 600 μl of methanol:water (1:1, v/v), and magnetic beads were added to the microcentrifuge tubes for homogenization using a homogenizer. Ultrasonic crushing was performed at a low temperature for 10 min, followed by −20°C for 30 min. The samples were then centrifuged at 14,500 rpm, 4°C for 15 min, and 200 μl of supernatant was dried in a vacuum centrifuge. Immediately afterward, the samples were redissolved with 200 µl of 50% methanol each and vortexed for 2 min. After ultrasonic crushing for 10 min at a low temperature, the microcentrifuge tube was centrifuged again at 14,500 rpm, 4°C for 15 min. Finally, the supernatant was stored in a sample injection bottle for analysis. Meanwhile, to prepare for the quality control (QC) sample, 100 μl of supernatant from each sample was taken in a 15-ml centrifuge tube in order to examine the stability and reproducibility of the entire analysis process. Frozen serum samples collected on BT1, AT1, and AT7 were thawed at 4°C, and vortexed for 2 min. For each sample, 200 μl of serum sample, 800 μl of methanol, and 10 μl of indole acetic acid ethyl ester (internal standard) were sequentially added to the 1.5-ml RNAase-free centrifuge tube and vortexed for 2 min. The samples were then centrifuged at 14,500 rpm, 4°C for 15 min; and 800 μl of supernatant was dried in a vacuum centrifuge for 3 h, blow-dried with nitrogen, and processed immediately. The next operation processes and QC sample preparation were similar to those of fecal samples.

### Multivariate Analysis

UPLC-Orbitrap-MS/MS analysis method was carried out as described previously (62), with minor modifications. The Compound Discoverer 2.1 (Thermo Fisher Scientific) data analysis tool was employed to automate complete raw data preprocessing and was applied to identify metabolites by searching the mzCloud library and mzVault library. In this study, MetaboAnalyst 5.0 (https://www.metaboanalyst.ca) was used to perform multivariate analysis. Principal component analysis (PCA) and orthogonal partial least-squares discriminant analysis (OPLS-DA) of metabolites were performed. Pathway enrichment analysis was performed by using the enrichment analysis module on MetaboAnalyst 5.0. The visualization results of the models were obtained with MetaboAnalyst 5.0.

### Statistical Analysis

SPSS 26.0 and GraphPad Prism 8.0 software were used for statistical analysis and graphical presentation. One-way ANOVA followed by the multiple range test of least significant difference was used to determine the statistical significance of multiple comparisons. All data were expressed as the mean ± standard error (SE). Significant differences were at *p* < 0.05, and tendencies were at *p* < 0.10. To preliminarily screen the differential metabolites, we selected the metabolites that had a *p-*value of less than 0.05 (calculated by Student’s t-test) and a variable importance in projection (VIP) score greater than 1.0 (calculated using Orthogonal PLS-DA model). Spearman’s correlation values and significance were computed with the R version 3.6.1. Clustering correlation heatmap with signs was performed using the OmicStudio tools at https://www.omicstudio.cn.

## Results

### Effect of Gallic Acid on Fecal Scores, Serum Hormone, HSP-70, Antioxidant Capacity, and Inflammatory Factors in Puppies

**Figure 2:**
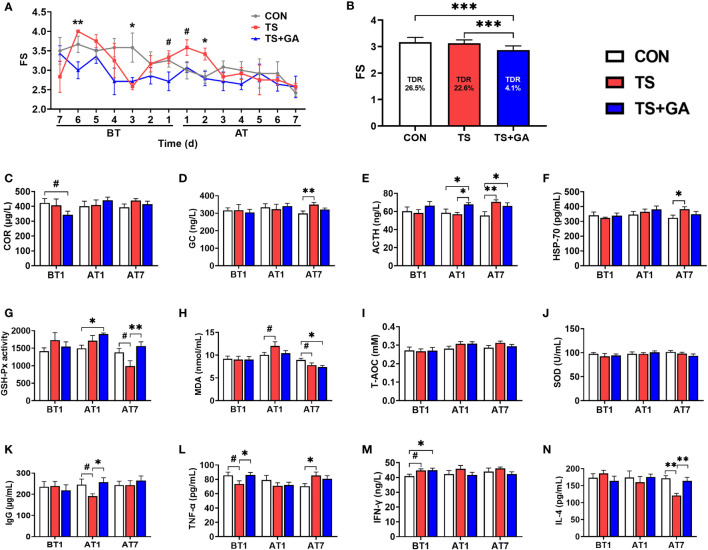
Effect of gallic acid (GA) on fecal score (FS) **(A, B)**, serum hormone **(C–E)**, HSP-70 **(F)**, antioxidant capacity **(G–J)**, and inflammatory factors **(K–N)** in puppies (n = 6 or 7). The symbol (*) indicates statistically significant differences between two groups (*p < 0.05, **p < 0.01, and ***p < 0.001), and the symbol (#) represents difference tendency (#*p* < 0.10). BT1, the 1st day before transportation; AT1, the 1st day after transportation; AT7, the 7th day after transportation. TDR (total diarrhea rate, %) = [cases of diarrhea during 14 days/(14 days × total puppies for each group)] × 100. COR, cortisol; ACTH, adrenocorticotropic hormone; GC, glucocorticoid; HSP-70, heat stress protein 70; GSH-Px, glutathione peroxidase; MDA, malondialdehyde; T-AOC, total antioxidant capacity; SOD, superoxide dismutase; IgG, immunoglobulin G; TNF-α, tumor necrosis factor-α; IFN-γ, interferon-γ; IL-4, interleukin 4.

Changes in FS are shown in **Figure 2A**. It is evident that the TS+GA group had lower FS than the CON or TS group on BT6 (*p* < 0.01), BT3 (*p* < 0.05), and BT1 (*p* = 0.085). And we found that FS increased in the TS group on AT1 (*p* = 0.085) and AT2 (*p* < 0.05). During the whole experimental period (**Figure 2B**), puppies fed GA (2.61 ± 0.05) had a normal fecal shape relative to the CON (3.17 ± 0.07) and TS groups (3.13 ± 0.07) (*p* < 0.001). Total diarrhea rate (TDR) in the CON, TS, and TS+GA groups were 26.5%, 22.6%, and 4.1%, respectively, and GA reduced TDR by as much as 84.5% and 81.9% compared with the CON and TS groups, respectively.

Puppies fed a diet containing 500 mg/kg of GA for 7 days had a trend toward lower COR compared with the CON group (*p* = 0.06, **Figure 2C**). Over time, there was no significant change. The TS group displayed a higher glucocorticoid (GC) level on AT7 (*p* < 0.01; **Figure 2D**). Similarly, ACTH acts on the adrenal cortex and stimulates GC and COR secretion; thus, it had a similar change as GC and COR (**Figure 2E**). No difference in HSP-70 was observed on BT1 and AT1 (**Figure 2F**); however, over time, the TS group had a higher HSP-70 level than the CON group on AT7 (*p* < 0.05), and puppies fed GA had no significant change as compared with the TS group.

There was no different GSH-Px activity between the CON and TS groups on BT1 and AT1 (**Figure 2G**), while puppies fed GA had higher GSH-Px activity than the CON group on AT1. And a decreasing trend of GSH-Px activity was observed in the TS group over the CON group on AT7 (*p* = 0.057). Dietary GA supplementation markedly improved the GSH-Px activity after transportation (*p* < 0.01). Additionally, the TS group had a marginally higher MDA level than the CON group on AT1 (*p* = 0.058, **Figure 2H**), whereas the TS group had a decreasing trend of MDA than the CON group (*p* = 0.061), and the TS+GA group had a decreasing MDA level over the CON group on AT7 (*p* < 0.05). The T-AOC and SOD contents had no obvious change among groups (**Figures 2I, J** **)**.

The TS group tended to decrease the serum IgG level on AT1 relative to the CON group (*p* = 0.093, **Figure 2K**), while puppies fed GA had a higher IgG level than the TS group (*p* < 0.05). Though both the CON and TS+GA groups had surprisingly higher TNF-α levels than the TS group on BT1 (*p* = 0.059, *p* < 0.05, **Figure 2L**), the TS group had a higher TNF-α level than the CON group over time on AT7 (*p* < 0.05), and no significant difference was observed between the TS+GA and TS groups. Similarly, the TS and TS+GA groups showed an unexpected increase in IFN-γ level over the CON group on BT1 (*p* = 0.053, *p* < 0.05, **Figure 2M**), but there was no difference among groups after transportation. Furthermore, IL-4 level sharply decreased in the TS group over the CON group on AT7 (*p* < 0.01, **Figure 2N**), while puppies fed GA had a significant increase of IL-4 level than the TS group (*p* < 0.01).

### Effect of Gallic Acid on Gut Microbial Composition and Structure in Puppies

**Figure 3:**
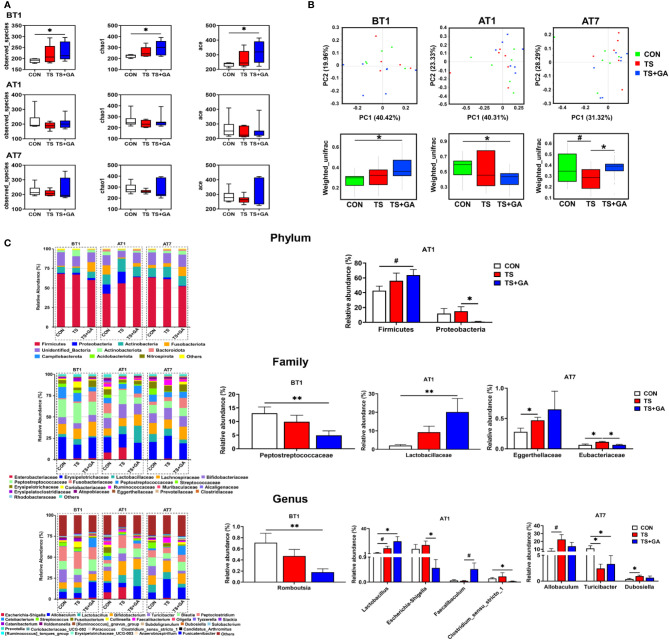
Effect of gallic acid (GA) on gut microbial composition and structure in puppies (n = 6 or 7). Alpha diversity **(A)**, principal coordinate analysis (PCoA) based on weighted UniFrac distances **(B)**, predominant fecal microbial communities, and different bacteria at the phylum, family, and genus levels **(C)**. The symbol (*) indicates statistically significant differences between two groups (*p < 0.05 and **p < 0.01), and the symbol (#) represents difference tendency (#*p* < 0.10). BT1, the 1st day before transportation; AT1, the 1st day after transportation; AT7, the 7th day after transportation.

On BT1, puppies fed a basal diet at 500 mg/kg of GA for 7 days had more Observed_species and higher Chao1 and ACE indices than those of the CON group (*p* < 0.05, **Figure 3A**). No difference was observed among the three groups on AT1 and AT7. From the difference of beta diversity index based on weighted UniFrac distances, PCoA plots revealed distinct separation between the CON and TS+GA groups on BT1 and AT1 (*p* < 0.05, **Figure 3B**), whereas the CON group had a trend toward significant separation relative to the TS group on AT7 (*p* = 0.095), especially that puppies fed the dietary supplementation of GA had distinct separation over the TS group (*p* < 0.05).

The most abundant phyla included Firmicutes (60.24%), Actinobacteria (11.47%), Fusobacterota (6.52%), Actinobacteriota (3.52%), Proteobacteria (3.24%), and Bacteroidota (1.13%) at various time points (**Figure 3C**). Puppies fed GA had the highest Firmicutes abundance on AT1 and tended to have higher Firmicutes than the CON group (*p* = 0.055). Furthermore, GA caused inhibition of Proteobacteria growth induced by transportation stress (*p* < 0.05). Also, the most abundant families included Erysipelotrichaceae (23.28%), Peptostreptococcaceae (12.53%), Lachnospiraceae (12.22%), Bifidobacteriaceae (11.36%), Peptostreptococcaceae (7.18%), Lactobacillaceae (6.60%), and Fusobacteriaceae (6.52%) at various phases. Decreasing Peptostreptococcaceae abundance was observed in the TS+GA group compared with the CON group on AT1 (*p* < 0.05). In contrast, a relative abundance of Lactobacillaceae was higher in the TS+GA group compared with the CON group on AT1 (*p* < 0.05). Relative abundance of Eggerthellaceae in the TS group significantly increased over the CON group, and the TS group had a higher Eubacteriaceae abundance than the CON and TS+GA groups (*p* < 0.05). Finally, the most abundant genera were *Allobaculum* (16.08%), *Bifidobacterium* (11.36%), *Peptoclostridium* (11.08%), *Blautia* (8.48%), *Lactobacillus* (6.60%), *Turicibacter* (5.26%), *Cetobacterium* (4.40%), *Escherichia–Shigella* (2.46%), *Streptococcus* (2.33%), *Fusobacterium* (2.08%), *Collinsella* (1.67%), and *Faecalibacterium* (1.27%). Relative abundance of *Romboutsia* significantly decreased in the TS+GA group compared with the CON group on BT1. Relative abundances of *Lactobacillus* and *Faecalibaculum* were higher, and relative abundances of *Escherichia–Shigella* and *Clostridium_sensu_stricto_1* were lower in the TS+GA group compared with the CON or TS group on AT1 (*p* < 0.05). The TS group had a higher relative abundance of *Allobaculum* and *Dubosiella* than the CON group on AT7 (*p* < 0.05), while no difference was observed in the TS+GA group; and both the TS and TS+GA groups had lower *Turicibacter* relative to the CON group (*p* < 0.05).

**Figure 4:**
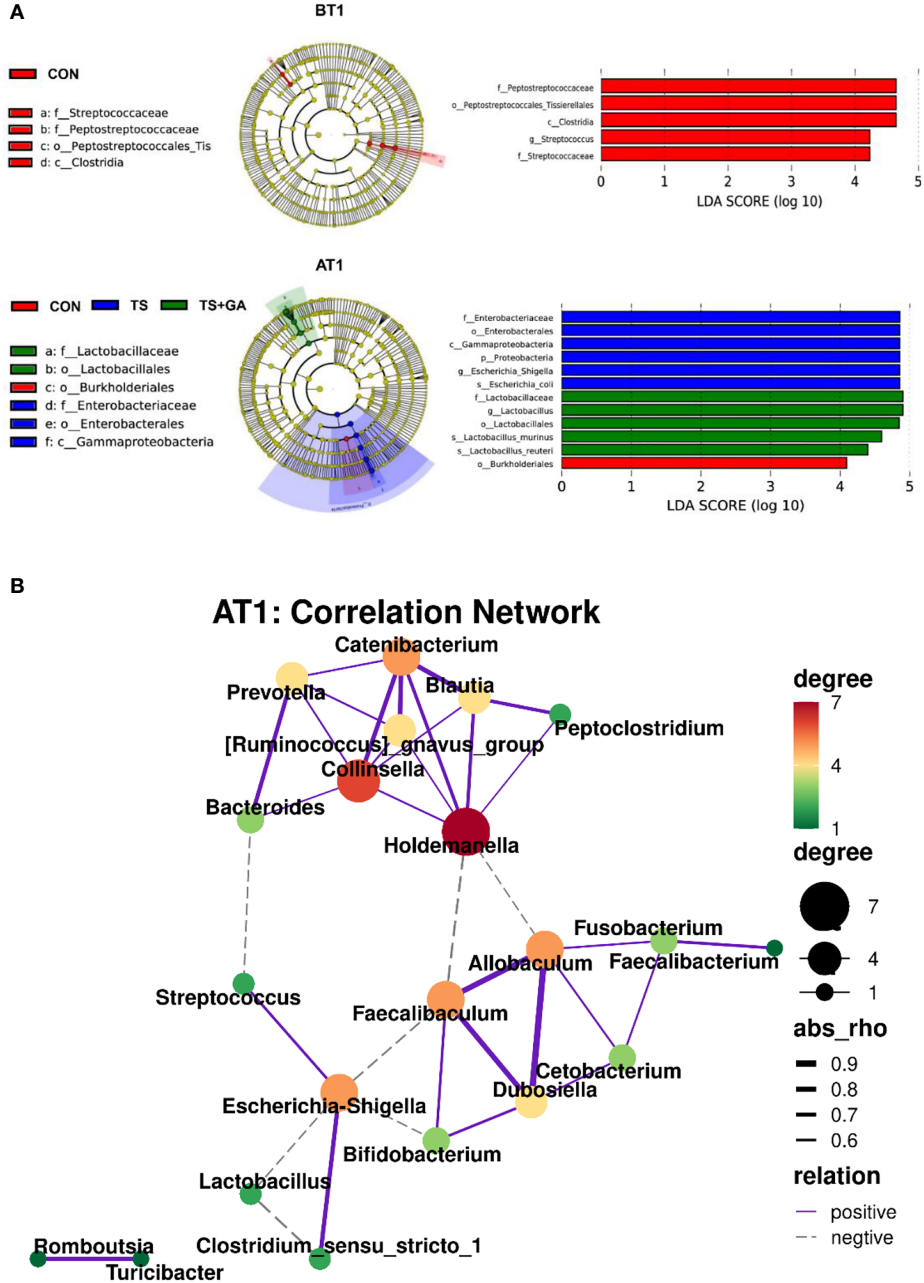
The linear discriminant analysis effect size (LEfSe) analysis identified gut bacterial biomarkers in puppies on BT1 and AT1 **(A)**. Spearman’s correlation network of fecal microbiota at genus level on AT1 (purple solid line, positive correlation; gray dotted line, negative correlation; thick line, significant correlation, *p* < 0.05) **(B)**. BT1, the 1st day before transportation; AT1, the 1st day after transportation.

Differential taxon abundances were further confirmed by LEfSe analysis. The histogram with logarithmic LDA score >4.0 and cladogram is shown in **Figure 4A**. On BT1, the LEfSe analysis indicated that Peptostreptococcaceae and *Streptococcus* in the CON group were the most abundant, whereas on AT1, the predominant bacterial strains in the TS group were *Escherichia*–*Shigella* and *Escherichia coli*. Fortunately, *Lactobacillus*, *Lactobacillus murinus*, and *Lactobacillus reuteri* were the highest in the TS+GA group, while no difference was observed on AT7. We next determined the relationship and interaction among fecal microbiota using Spearman’s correlation analysis. As shown in **Figure 4B**, *Escherichia–Shigella* negatively modulated *Faecalibaculum*, *Lactobacillus*, and *Bifidobacterium* and positively modulated *Streptococcus* and *Clostridium_sensu_stricto_1* in the network. *Allobaculum* positively modulated *Faecalibaculum*, *Dubosiella*, *Cetobacterium*, and *Fusobacterium*. There was a positive network among *Catenibacterium*, *Prevotella*, *Collinsella*, *[Ruminococcus]_gnavus_group*, *Holdemanella*, *Blautia*, and *Peptoclostridium*. In addition, we also found a positive correlation between *Romboutsia* and *Turicibacter*. Regarding Spearman’s analysis, the whole network of microbiota was divided into several parts, in which genera *Escherichia–Shigella*, *Allobaculum*, *Catenibacterium*, and *Holdemanella* dominated key positions and had close interactions with many bacteria in the community.

The gut bacterial function and pathways after transportation and GA treatment were predicted by PICRUSt analysis based on the Kyoto Encyclopedia of Genes and Genomes (KEGG) pathway using 16S rRNA data. On BT1, puppies fed GA had more abundant amino acid metabolism, energy metabolism, carbohydrate metabolism, nucleotide metabolism, metabolism of cofactors and vitamins, and metabolism of terpenoids and polyketide (**Figure S1A**), indicating that these metabolic pathways were significantly influenced by GA in the short term. It is worth noting that the decreasing abundance of genes involved in energy metabolism and glycan biosynthesis and metabolism were found in the TS+GA group relative to the CON group on AT1 (**Figure S1B**), while more abundant carbohydrate metabolism was observed in the TS+GA group over the TS group. On AT7, puppies transported to another livable environment had weaker amino acid metabolism and xenobiotics biodegradation and metabolism than the CON group (**Figure S1C**), whereas energy metabolism, xenobiotics biodegradation and metabolism, and metabolism of cofactors and vitamins were markedly enhanced after GA treatment compared with those of the CON group.

### Effect of Gallic Acid on Fecal Short-Chain Fatty Acids and Branched-Chain Fatty Acids in Puppies

**Figure 5:**
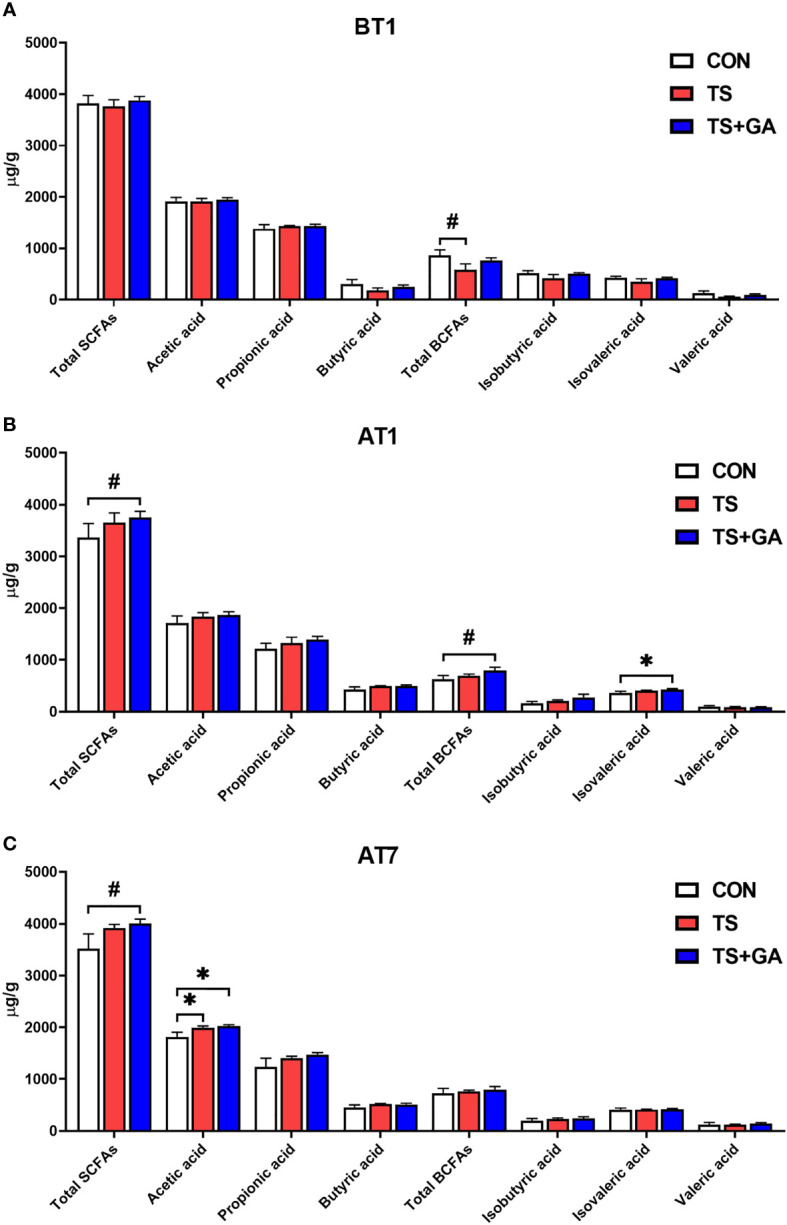
Effect of gallic acid (GA) on fecal short-chain fatty acids (SCFAs) and branched-chain fatty acids (BCFAs) in puppies on BT1 **(A)**, AT1 **(B)**, and AT7 **(C)** (n = 6 or 7). The symbol (*) indicates statistically significant differences between two groups (*p < 0.05), and the symbol (#) represents difference tendency (#*p* < 0.10). BT1, the 1st day before transportation; AT1, the 1st day after transportation; AT7, the 7th day after transportation.

No significant differences in SCFAs concentrations were observed among the three groups except for total BCFAs between the CON and TS groups on BT1 (*p* = 0.057; **Figure 5A**), while puppies fed GA had a trend of increase in total SCFAs (*p* = 0.083; **Figure 5B**) and increasing total BCFAs (*p* = 0.087) and isovaleric acid (*p* < 0.05) content relative to the CON group on AT1. Similarly, the TS+GA group had a similar trend of increase in total SCFAs to the CON group (*p* = 0.099; **Figure 5C**), and higher acetic acid levels were observed in the TS and TS+GA groups in comparison with the CON group on AT7 (*p* < 0.05).

### Effect of Gallic Acid on Fecal Metabolites in Puppies

**Figure 6:**
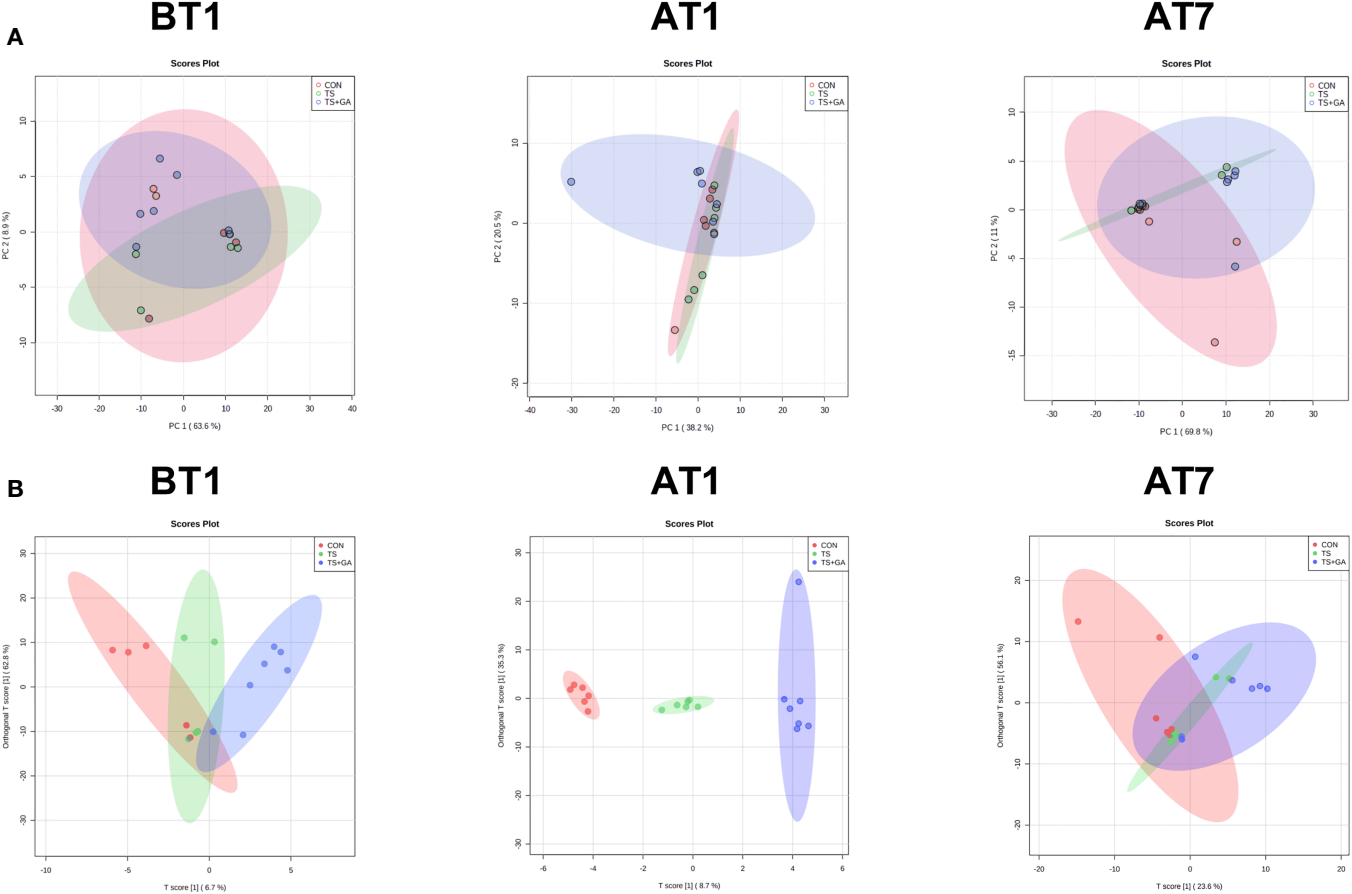
Multivariate statistical analysis on BT1, AT1, and AT7 (n = 6 or 7). Score plots from the principal component analysis (PCA) model among three groups on BT1, AT1, and AT7 **(A)**. Score plots from the orthogonal partial least-squares discriminant analysis (OPLS-DA) model among three groups on BT1, AT1, and AT7 **(B)**. BT1, the 1st day before transportation; AT1, the 1st day after transportation; AT7, the 7th day after transportation.

Multivariate statistical analysis was carried out among three groups. In this study, the PCA was used to study the differences among the CON, TS, and TS+GA groups in the fecal metabolomics by an unsupervised statistical method (**Figure 6A**). The PCA score plots showed less obvious separation at varying time points. However, the OPLS-DA model revealed a clearer difference between the three clusters on AT1 (**Figure 6B**), indicating that the difference among the three groups was the most obvious when puppies were transported from a stressful environment to another livable location.

**Figure 7:**
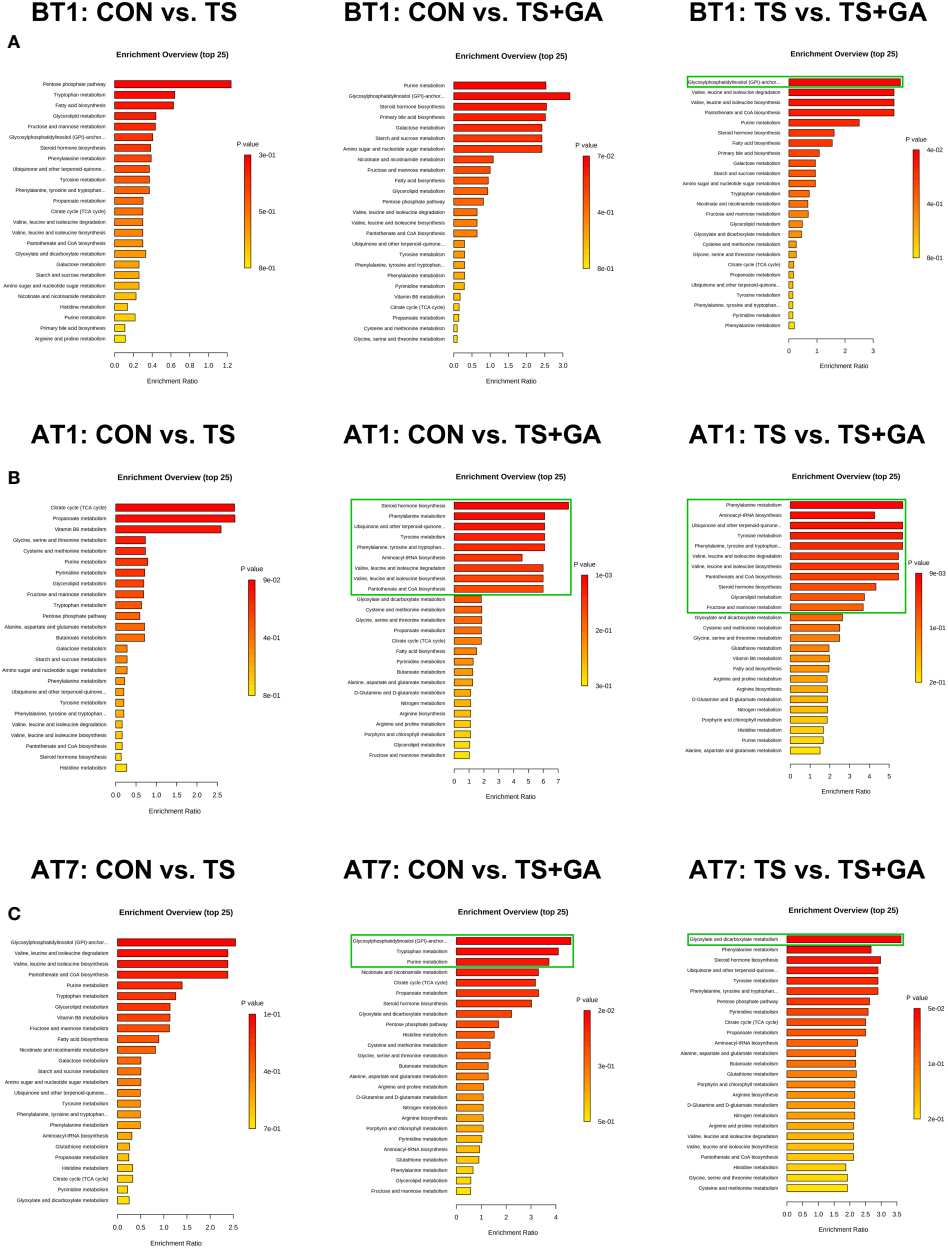
Bar charts of the metabolic pathway analysis of differential fecal metabolites on BT1 **(A)**, AT1 **(B)**, and AT7 **(C)** (n = 6 or 7). The pathway enrichment analysis shows all matched pathways, and the green boxes indicate significant metabolic pathways (*p* < 0.05). BT1, the 1st day before transportation; AT1, the 1st day after transportation; AT7, the 7th day after transportation.

In this study, a total of 156 metabolites were detected at all stages (**Table S2**). The differential metabolites at varying time points are shown in **Table S3**. A total of 6, 16, and 8 potential biomarkers were identified on BT1, AT1, and AT7, respectively. To gain further insight into the metabolic changes, a KEGG pathway analysis of all metabolites was performed. On BT1, the influenced pathway was mainly concentrated in glycan biosynthesis and metabolism (glycosylphosphatidylinositol (GPI)-anchor biosynthesis) (**Figure 7A**). On AT1, the most influenced metabolic pathways were amino acid metabolism (phenylalanine metabolism, tyrosine metabolism; phenylalanine, tyrosine, and tryptophan biosynthesis; valine, leucine, and isoleucine degradation; and valine, leucine, and isoleucine biosynthesis), lipid metabolism (steroid hormone biosynthesis and glycerolipid metabolism), metabolism of cofactors and vitamins (ubiquinone and other terpenoid-quinone biosynthesis, and pantothenate and CoA biosynthesis), and carbohydrate metabolism (fructose and mannose metabolism) (**Figure 7B**). On AT7, the most important metabolic pathways were carbohydrate metabolism (purine metabolism, and glyoxylate and dicarboxylate metabolism), amino acid metabolism (tryptophan metabolism), and glycan biosynthesis and metabolism (GPI-anchor biosynthesis) (**Figure 7C**). As a result, we found that the significant differences in the metabolic pathways were mainly concentrated in AT1. The levels of predominant potential biomarkers based on the significant metabolic pathways on BT1, AT1, and AT7 are shown in **Table S4**.

### Effect of Gallic Acid on Serum Metabolites in Puppies

**Figure 8:**
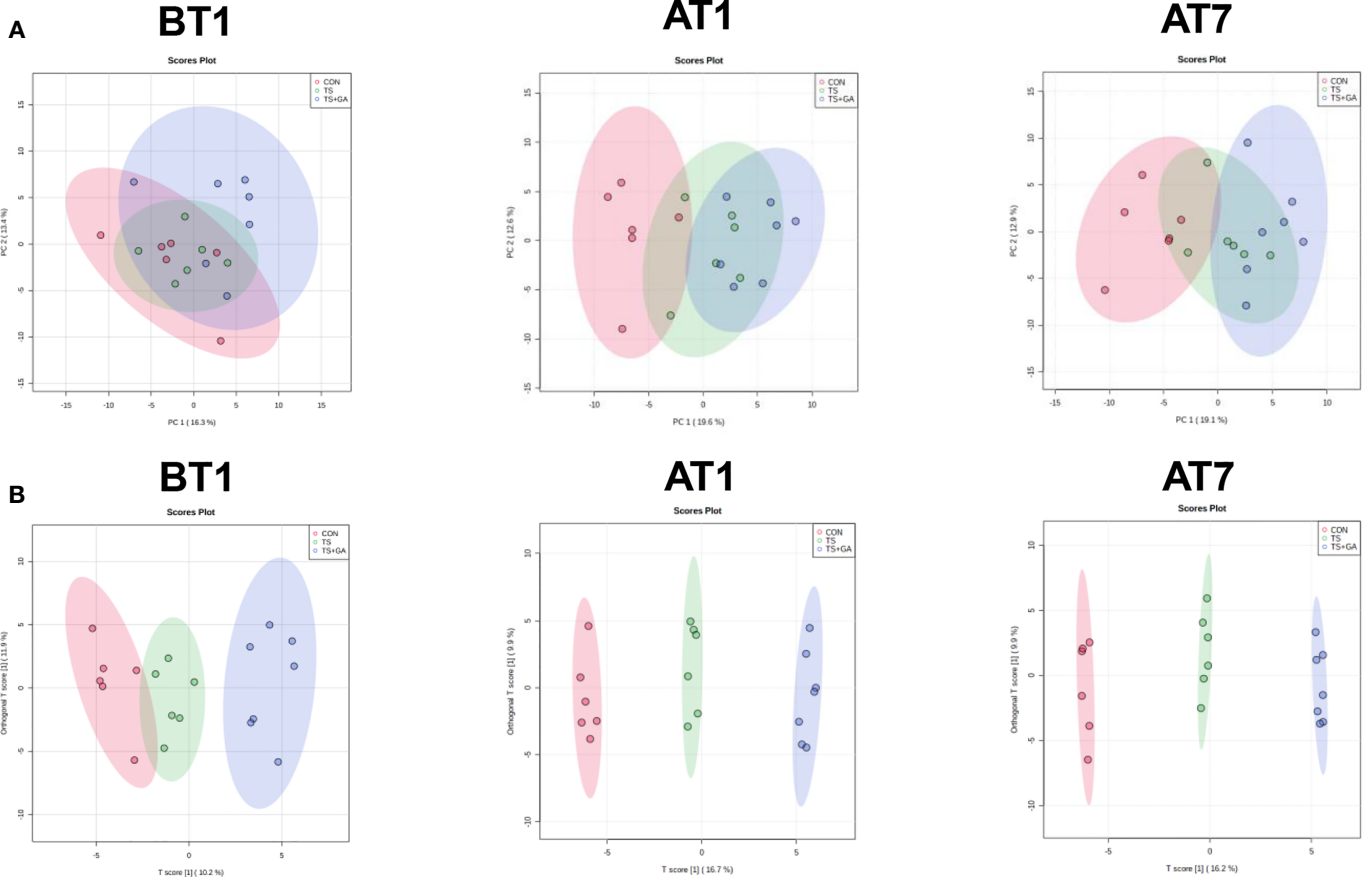
Multivariate statistical analysis on BT1, AT1, and AT7 (n = 6 or 7). Score plots from the principal component analysis (PCA) model among three groups on BT1, AT1, and AT7 **(A)**. Score plots from the orthogonal partial least-squares discriminant analysis (OPLS-DA) model among three groups on BT1, AT1, and AT7 **(B)**. BT1, the 1st day before transportation; AT1, the 1st day after transportation; AT7, the 7th day after transportation.

Based on the fecal metabolomics analysis, we further detected serum metabolomics. As shown in **Figure 8A**, the PCA score plots showed distinct separation among the CON, TS, and TS+GA groups after transportation. Similarly, the score plots for the OPLS-DA model presented clear separation over time (**Figure 8B**), suggesting a difference among the three groups. From these results of multivariate statistical analysis, it is apparent that there are greater differences in serum metabolites than fecal metabolites at different stages.

**Figure 9:**
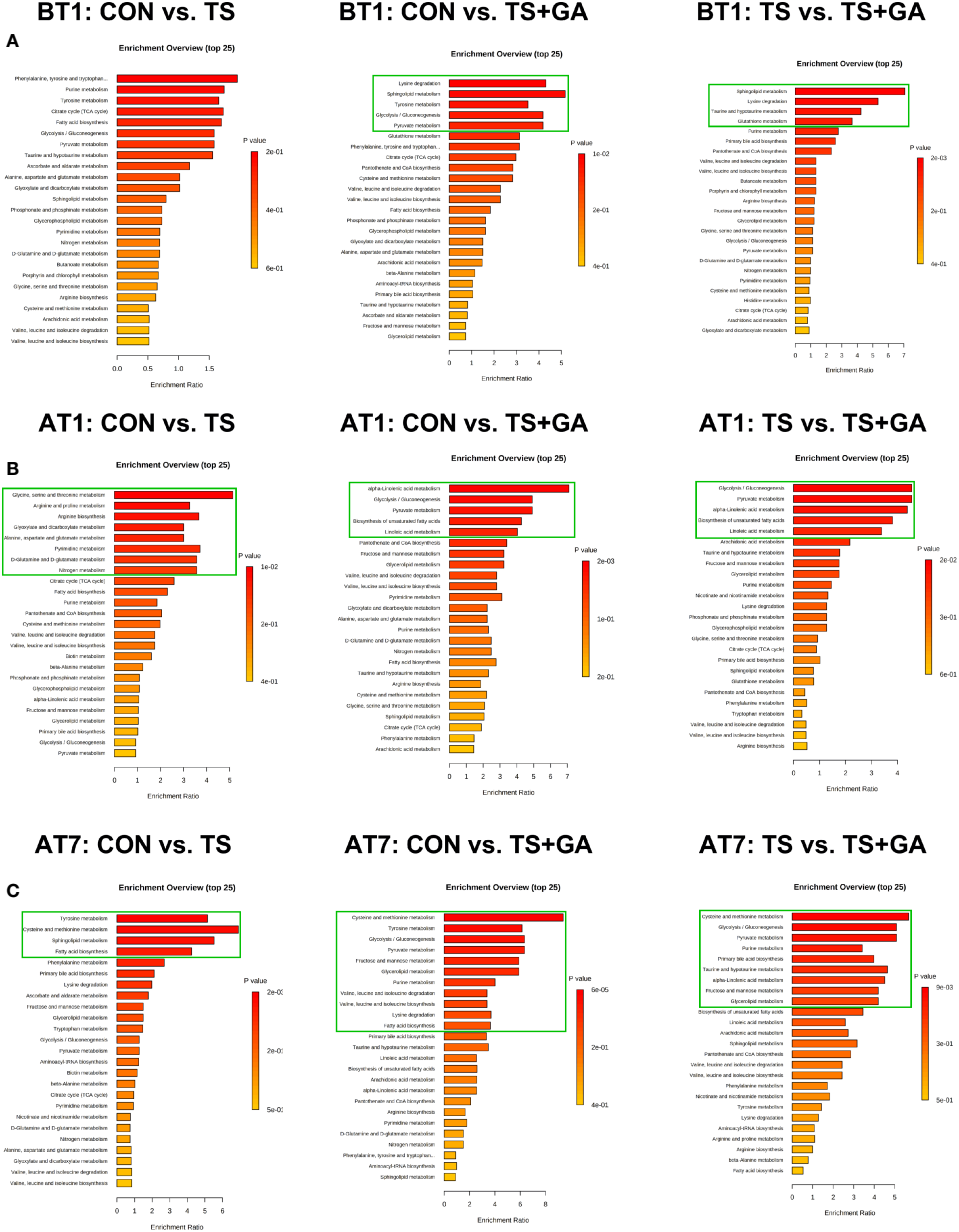
Bar charts of the metabolic pathway analysis of differential serum metabolites on BT1 **(A)**, AT1 **(B)**, and AT7 **(C)** (n = 6 or 7). The pathway enrichment analysis shows all matched pathways, and the green boxes indicate significant metabolic pathways (*p* < 0.05). BT1, the 1st day before transportation; AT1, the 1st day after transportation; AT7, the 7th day after transportation.

In this study, a total of 147 metabolites were detected at all stages (**Table S5**). The differential metabolites at varying time points are shown in **Table S6**. A total of 13, 48, and 36 potential biomarkers were identified on BT1, AT1, and AT7, respectively. On BT1, puppies fed GA mainly influenced serum amino acid metabolism (lysine degradation, tyrosine metabolism, taurine and hypotaurine metabolism, and glutathione metabolism), carbohydrate metabolism (glycolysis/gluconeogenesis and pyruvate metabolism), and lipid metabolism (sphingolipid metabolism) (**Figure 9A**). On AT1, the influenced pathway was mainly concentrated in amino acid metabolism (glycine, serine, and threonine metabolism; arginine and proline metabolism; arginine biosynthesis, alanine, aspartate, and glutamate metabolism; and d-glutamine and d-glutamate metabolism), carbohydrate metabolism (glyoxylate and dicarboxylate metabolism), energy metabolism (nitrogen metabolism), and nucleotide metabolism (pyrimidine metabolism) between the CON and TS groups (**Figure 9B**), whereas feeding GA was implicated in the regulation of carbohydrate metabolism (glycolysis/gluconeogenesis and pyruvate metabolism) and lipid metabolism (alpha-linolenic acid metabolism, linoleic acid metabolism, and biosynthesis of unsaturated fatty acids) compared with the other two groups. On AT7, the affected pathways mainly involved amino acid metabolism (tyrosine metabolism and cysteine and methionine metabolism) and lipid metabolism (sphingolipid metabolism and fatty acid biosynthesis) in the TS group compared with the CON group (**Figure 9C**); notably, significant enrichment of several major metabolic pathways, such as amino acid metabolism (cysteine and methionine metabolism; tyrosine metabolism; valine, leucine, and isoleucine degradation; valine, leucine, and isoleucine biosynthesis; lysine degradation; and taurine and hypotaurine metabolism), carbohydrate metabolism (glycolysis/gluconeogenesis, pyruvate metabolism, and fructose and mannose metabolism), lipid metabolism (glycerolipid metabolism, fatty acid biosynthesis, primary bile acid biosynthesis, and alpha-linolenic acid metabolism), and nucleotide metabolism (purine metabolism), was significantly changed by GA. The levels of predominant potential biomarkers based on the significant metabolic pathways on BT1, AT1, and AT7 were presented in **Table S7**.

### The Correlation Analysis of Metabolites and Microbiota

**Figure 10:**
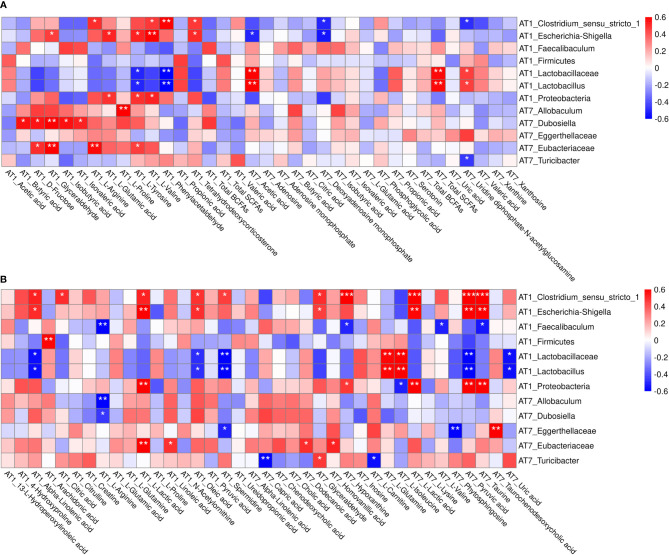
Spearman’s correlation analysis between the differential feces metabolites and fecal microbiota **(A)**, and the differential serum metabolites and fecal microbiota **(B)** on AT1 and AT7. The symbol (*) indicates a significant association between metabolite and microbiota (*P < 0.05, **P < 0.01, and ***P < 0.001). Red color indicates a positive correlation, and blue color indicates a negative correlation. AT1, the 1st day after transportation; AT7, the 7th day after transportation.

Spearman’s correlation analysis was performed for the differential feces and serum metabolites and fecal microbiota obtained by high-throughput 16S rRNA sequencing. On AT1, we found that fecal l-arginine, l-valine, phenylacetaldehyde, and tetrahydrodeoxycorticosterone were positively correlated with the relative abundance of *Clostridium_sensu_stricto_1* **(** **Figure 10A**). And glyceraldehyde, l-glutamic acid, l-tyrosine, l-valine, and tetrahydrodeoxycorticosterone were positively correlated with the relative abundance of *Escherichia–Shigella*. l-Glutamic acid, l-tyrosine, and l-valine were also positively correlated with Proteobacteria. Conversely, l-tyrosine and phenylacetaldehyde were negatively correlated with Lactobacillaceae and *Lactobacillus*. In addition, we also observed a weak positive association between *Faecalibaculum* with total BCFAs (isobutyric acid and isovaleric acid), and the total SCFAs (acetic acid and propionic acid) had a weak positive association with Firmicutes. On AT7, uridine diphosphate-*N*-acetylglucosamine was negatively correlated with the relative abundance of *Turicibacter*. Furthermore, butyric acid and isobutyric acid had a weak positive association with the *allobaculum*.

As shown in **Figure 10B**, alpha-linolenic acid, citric acid, l-lactic acid, oleic acid, and spermidine were positively correlated with *Clostridium_sensu_stricto_1* on AT1. Likewise, alpha-linolenic acid, l-lactic acid, and oleic acid were also positively correlated with *Escherichia–Shigella*. And a significant positive association was found between l-lactic acid with Proteobacteria. In contrast, alpha-linolenic acid, oleic acid, and spermidine were negatively correlated with Lactobacillaceae and *Lactobacillus*. Additionally, strong negative and positive associations of the l-arginine with *Faecalibaculum* and the arachidonic acid with Firmicutes were observed. On AT7, serum phytosphingosine and taurochenodeoxycholic acid had a reverse association with Eggerthellaceae. Similarly, glyceraldehyde and l-carnitine had positive and negative associations with *Turicibacter*. And dodecanoic acid and homovanillic acid were positively correlated with Eubacteriaceae.

## Discussion

To date, adequate evidence exists to support the antioxidation, anti-inflammatory, and antimicrobial activities of GA (45, 63, 64). In this study, we summarized the results of 16S rRNA gene sequencing and metabolomics analysis and discussed the effects of environmental stress and GA on the host-microbial metabolic axis from the relationship between metabolic biomarkers and gut bacteria. Our results suggested that dietary GA supplementation reduced multiple stress-induced diarrhea in puppies by enhancing systemic and intestinal defenses. The diarrhea rate still seemed to be high over a short period, and a possible reason was the GM disturbance induced by stress, whereas puppies fed GA maintained normal FS and lower diarrhea rate by inhibiting the growth of pathogenic bacteria *Escherichia–Shigella* throughout the experiment. Unexpectedly, FS in the TS group fluctuated considerably before transportation, whose reason may be the large individual differences for all puppies in response to the stressful environment. A previous study conducted by Cai et al. (50) confirmed that dietary GA supplementation at 400 mg/kg reduced diarrhea incidence in weaned piglets.

Meanwhile, stress activates the HPA axis and triggers a cascade of hormonal release (65, 66). Aligned with previous studies, the present study showed an increasing serum ACTH level after transportation, indicating that stress activates the hypothalamus to secrete CRH and induces the anterior pituitary gland to release ACTH. The ACTH acts on the adrenal cortex to produce GC COR, which negatively regulates CRH production to terminate the stress response cascade. However, dietary supplementation with 500 mg/kg of GA resulted in lower serum COR, GC, and ACTH levels on day 7 after transportation, indicating that GA has great potential to relieve stress. High HSP-70 level is also induced by inflammatory stress and oxidative stress except for heat shock (67–69). Our results revealed that puppies in the TS group had higher HSP-70 levels after transportation, which were consistent with increased inflammatory response (TNF-α↑, IL-4↓) and oxidative stress (GSH-Px↓) caused by transportation and changing environment, while GA suppressed upregulation of HSP-70 level. Similarly, studies on other polyphenol compounds in animals also obtained similar results (70, 71).

Previous studies revealed that the addition of dietary GA could modulate different signaling pathways through a wide range of inflammatory cytokines and enzymatic and non-enzymatic antioxidant defense systems (72). The enzymatic antioxidant defense system is generally the primary line of antioxidant defense in ROS detoxification (73). Additionally, MDA is the principal end-product of the lipid peroxidation process (74). So far, several studies reported the antioxidant action of GA (64, 75), and GA could provide the protection for various potential diseases including cancer, cardiovascular disease, and metabolic disease under oxidative stress by restoring the lipid peroxidation levels, normalizing or enhancing the levels of SOD, CAT, GSH-Px, GST, and GSH (76–78). In the present study, we found that multiple stressors resulted in a significant decrease in serum GSH-Px activities and an increase in MDA production in puppies. Nevertheless, dietary supplementation with GA at 500 mg/kg protected puppies from oxidative damage by increasing the activity of serum GSH-Px, which can efficiently eliminate free radicals and reduce the synthesis of MDA. Our results were consistent with those described in other studies.

Cytokines also play an important role in the regulation of intestinal function (79), while the overproduction of proinflammatory cytokines has a negative influence on intestinal homeostasis (80). It has been reported that the release of the pro-/anti-inflammatory and inflammatory mediators, such as IL-2, IL-4, IL-5, IL-13, IL-33, TNF-α, IFN-γ, and NF-κB, could be downregulated by GA to prevent excessive inflammatory responses (41, 81, 82). Similarly, our results indicated that environmental stress caused a systemic inflammatory response by decreasing serum IgG content, increasing the production of proinflammatory cytokines TNF-α and IFN-γ, and reducing the secretion of anti-inflammatory cytokine IL-4 contents. However, GA effectively reversed the inflammatory responses in puppies, indicating that 500 mg/kg of dietary GA can improve anti-inflammatory function in stressed puppies. Also, a recent review concluded that GA plays an anti-inflammatory role by modulating the GM (40).

Previous studies indicated that GA was effective in a broad spectrum of antibacterial applications against pathogens including *E. coli*, *Staphylococcus aureus*, *Pseudomonas aeruginosa*, *Klebsiella pneumonia*, *Streptococcus mutans*, *Chromobacterium violaceum*, *Campylobacter jejuni*, and *Listeria monocytogenes* (83–85). Our study also reached a similar inhibitory effect on pathogenic bacteria. Dietary supplementation with 500 mg/kg of GA improved the bacterial diversity, inhibited the growth of *Escherichia*–*Shigella* and *Clostridium_sensu_stricto_1*, and enhanced *Lactobacillus* and *Faecalibaculum*, especially 1 day after transportation. Our results were further verified with LEfSe analysis, which also found that the TS group was associated with enrichment of Proteobacteria, *Escherichia*–*Shigella*, and *E. coli*, while *Lactobacillus*, *L. murinus*, and *L. reuteri* dominated the GA treatment (86, 87). Spearman’s correlation analysis revealed the symbiotic relationship between bacteria. The high coexistence of *Escherichia–Shigella* with *Streptococcus* and *Clostridium_sensu_stricto_1* suggested the possibility of a syntrophic relationship among these bacteria. However, *Escherichia–Shigella* negatively correlated with *Faecalibaculum*, *Lactobacillus*, and *Bifidobacterium*. In agreement, the high relative abundance of pathogen *Escherichia–Shigella* is reported to be accompanied by the low relative abundance of *Lactobacillus* (88, 89). Our results are in general agreement with the previous studies, which found a decrease in Lactobacillaceae and Prevotellaceae and an increase in Firmicutes and Proteobacteria phyla in dextran sodium sulfate-induced colitis in mice, and GA treatment could modulate the microbiota composition toward a similar proportion to the control group (45, 90). Furthermore, Lima et al. reported that *Escherichia–Shigella* is one of the leading pathogenic causes of diarrhea, affecting approximately 80–165 million individuals (91). We can therefore infer that GA has a potential prophylactic effect on diarrhea caused by *Escherichia–Shigella*. GA also can induce changes in the microbiota toward a more favorable composition and activity, including the production of SCFAs and BCFAs in the colon (90).

The digestive tract contains an abundance of gut microbiota-derived metabolites (92). As one of the most important microbiota-derived metabolites, SCFAs are generated through colonic fermentation of dietary fibers (93, 94) and exert a beneficial effect on host health by reducing colonic pH and inflammation (95, 96), stimulating enterocyte growth, and improving mucus production and epithelial health (97). A previous study showed that increases in fecal SCFAs were found when relative abundances of Firmicutes, Lactobacillaceae, Clostridiales, *Roseburia*, Lachnospiraceae, and Erysipelotrichaceae were increased (98). These studies were in accordance with our findings that dietary GA treatment led to the increment of fecal total SCFAs and acetic acid concentrations. Further Spearman’s correlation analysis revealed that fecal SCFAs have a positive association with Firmicutes (Erysipelotrichaceae, *Faecalibaculum*, *Allobaculum*, *Turicibacter*, and *Dubosiella*) and Lactobacillaceae (*Lactobacillus*) after transportation. Fecal BCFAs (e.g., isobutyric, isovaleric acid, valeric acid) are generated by microbial fermentation of branched amino acids, valine, leucine, and isoleucine (99, 100) and have effects on lipid and glucose metabolism (101). The highest total BCFAs and isovaleric acid concentrations were observed in the TS+GA group at 1 day after transportation, which had a positive association with *Faecalibaculum*, indicating that BCFAs may be produced by *Faecalibaculum*. The conclusion needs further validation. In short, these results indicate that GA protects against environmental stress-induced inflammation by improving the intestinal microbial structure and increasing the relative abundance of SCFA-producing bacteria.

Microbiota-derived metabolites, often secreted in the intestine and translocated across the intestinal barrier into the circulating system, are very important modulators for host metabolism (102, 103). In our study, metabolomics based on UPLC-Orbitrap-MS/MS analysis method was applied to investigate the changes of fecal metabolites in beagle dogs. The KEGG enrichment analysis declared that environmental stress mainly disturbed amino acid metabolism, carbohydrate metabolism, lipid metabolism, and metabolism of cofactors and vitamins in puppies, while dietary intake of GA helped to restore this imbalance. Changes in the metabolic pathway were consistent with the PICRUSt analysis. Our findings were largely similar to the results reported by the previous study, whose metabolic data revealed that the GA-induced feces and urine metabolic changes in mice mainly focus on increasing carbohydrate metabolism (gluco-related metabolism) and lipid metabolism (bile acid metabolism) and decreasing amino acid metabolism (45). By screening differential metabolites in major differential metabolic pathways, fecal phenylacetaldehyde, l-tyrosine, l-valine, serotonin (amino acid metabolism), xanthine, adenosine, xanthosine, uric acid, phosphoglycolic acid (carbohydrate metabolism), tetrahydrodeoxycorticosterone, and glyceraldehyde (lipid metabolism) were upregulated due to the GA treatment. We considered them as the biomarkers for evaluating the influence of dietary GA treatment on fecal metabolites in puppies.

4-*O*-Methygallic acid (4-OMeGA) is the primary metabolite of GA in human plasma and urine (104–106). The current study detected high levels of 4-OMeGA in the serum of puppies, indicating that GA may exert its function mainly by further transforming to 4-OMeGA. Serum metabolomics revealed that environmental stress mainly influenced amino acid metabolism, carbohydrate metabolism, lipid metabolism, energy metabolism, and nucleotide metabolism, while puppies fed GA reversed the shift. This finding is similar to that of Shi et al. who reported that metabolic changes associated with GA intake include glycogenolysis, glycolysis, tricarboxylic acid (TCA) cycle, and metabolism of nucleotides, choline, bile acids, amino acids (107). Consistent with fecal biomarkers analysis, serum metabolites of l-arginine, creatine, spermidine, 4-hydroxyproline, l-proline, l-glutamic acid, pyruvic acid, *N*-acetylornithine, citrulline, l-glutamine, l-valine, l-isoleucine, l-lysine, carnitine (amino acid metabolism), citric acid, l-lactic acid, glyceraldehyde (carbohydrate metabolism), alpha-linolenic acid, oleic acid, linoleic acid, arachidonic acid, 13-l-hydroperoxylinoleic acid, chenodeoxycholic acid, taurine, cholic acid, taurochenodeoxycholic acid (lipid metabolism), ureidopropionic acid, hypoxanthine, inosine, and uric acid (nucleotide metabolism) were chosen as the biomarkers for evaluating the influence of dietary GA treatment on serum metabolites in puppies.

Spearman’s correlation analysis found that fecal l-valine, l-tyrosine, l-glutamic acid, phenylacetaldehyde, and tetrahydrodeoxycorticosterone were positively correlated with the relative abundance of *Clostridium_sensu_stricto_1* (Firmicutes) and *Escherichia–Shigella* (Proteobacteria). However, interestingly, l-tyrosine and phenylacetaldehyde were oppositely correlated with and *Lactobacillus* (Lactobacillaceae). Serum l-lactic acid, alpha-linolenic acid, citric acid, oleic acid, and spermidine were positively correlated with *Clostridium_sensu_stricto_1* and *Escherichia–Shigella*, whereas alpha-linolenic acid, oleic acid, and spermidine were negatively correlated with *Lactobacillus* (Lactobacillaceae). Simultaneously, the positive correlation between serum metabolites and bacteria were l-arginine (*Faecalibaculum*), phytosphingosine and taurochenodeoxycholic acid (Eggerthellaceae), l-carnitine (*Turicibacter*), and dodecanoic acid and homovanillic acid (Eubacteriaceae); and serum arachidonic acid and glyceraldehyde had a positive association with Firmicutes and *Turicibacter*, respectively. Further research is needed to provide a clear explanation between GM and fecal and serum metabolome in puppies supplemented with GA.

## Conclusion

The GA markedly reduced the incidence of diarrhea and alleviated multiple environmental stressor-induced oxidative stress and inflammatory responses in puppies. The microbiome and metabolomics analyses revealed that environmental stress caused intestinal microbiota and metabolic disorders, while GA reversed the abnormalities. The comprehensive microbiota and metabolite relationships were established. In summary, we systematically elucidated the beneficial effects of GA treatment on stressed dogs from the host-microbial metabolic axis point of view. Future studies that can focus on the interactions between microbiota and metabolites may prove efficacious for understanding the precise mechanisms of the beneficial effects of polyphenol on health.

## Data Availability Statement

The datasets presented in this study can be found in online repositories. The names of the repository/repositories and accession number(s) can be found below: https://www.ncbi.nlm.nih.gov/bioproject/PRJNA782241.

## Ethics Statement

The animal study was reviewed and approved by the Experimental Animal Ethics Committee of South China Agricultural University.

## Author Contributions

KY generated the ideas, designed the study, detected the samples, and wrote the initial manuscript. LinZ and BD guided and revised the manuscript. XD and SJ participated in the data analysis and contributed to the draft of the manuscript. JD made feasible suggestions for the experimental design and manuscript. MZ analyzed the results. CW, ZQX, LimZ, AT, SY, PL, ZLX, SH, and FZ detected the samples. All authors contributed to the article and approved the submitted version.

## Funding

This project was supported by the National Natural Science Foundation of China (Grant Nos. 31790411 and 32002186), Natural Science Foundation of Guangdong Province (Grant No. 2020A1515010322), Guangdong Basic and Applied Basic Research Foundation (2019B1515210002), and Independent Research and Development Projects of Maoming Laboratory (2021ZZ003).

## Conflict of Interest

AT is employed by Guangzhou Qingke Biotechnology Co., Ltd.

The remaining authors declare that the research was conducted in the absence of any commercial or financial relationships that could be construed as a potential conflict of interest.

## Publisher’s Note

All claims expressed in this article are solely those of the authors and do not necessarily represent those of their affiliated organizations, or those of the publisher, the editors and the reviewers. Any product that may be evaluated in this article, or claim that may be made by its manufacturer, is not guaranteed or endorsed by the publisher.
