# Supplementary material for: Gallic Acid Alleviates Gut Dysfunction and Boosts Immune and Antioxidant Activities in Puppies Under Environmental Stress Based on Microbiome–Metabolomics Analysis
Source: Front Immunol. 2022 Jan 14;12:813890. doi: 10.3389/fimmu.2021.813890 (PMC8795593; doi:10.3389/fimmu.2021.813890)
Supplement: Supplementary file 1 [file DataSheet_1.docx]

Supplementary Material


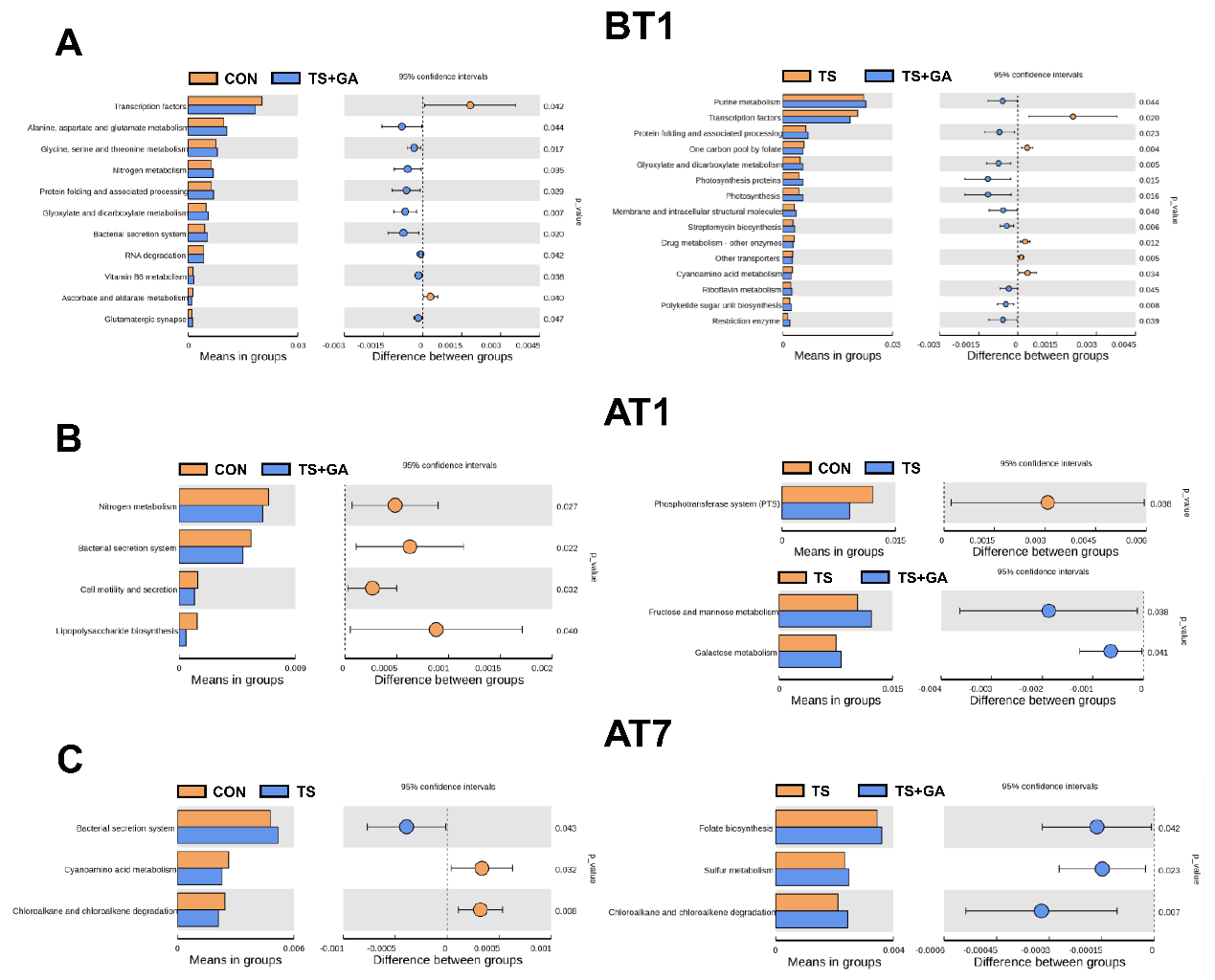


**FIGURE S1 |** Function prediction of gut bacterial community at level III. PICRUSt analysis on BT1 (**A**), AT1 (**B**), and AT7 (**C**). BT1 = the 1^st^ day before transportation; AT1 = the 1^st^ day after transportation; AT7 = the 7^th^ day after transportation.

**TABLE S1 |** The serum biochemistry and blood routine examination in test puppies one day before the trial.

| **Items** | **CON** | **TS** | **TS+GA** | **Reference range** |
| --- | --- | --- | --- | --- |
| **Serum biochemistry^1^** |  |  |  |  |
| ALT (U/L) | 42.00±3.92 | 50.17±5.06 | 44.14±2.55 | 5~125 |
| AST (U/L) | 35.50±2. 69 | 32.00±1.65 | 34.86±0.96 | 0~50 |
| TP (g/L) | 52.68±0.76 | 51.10±0.92 | 51.20±1.12 | 52.0~82.0 |
| ALB (g/L) | 25.73±0.52 | 25.65±0.68 | 25.93±0.44 | 23~40 |
| ALP (U/L) | 156.50±9.91 | 166.83±21.16 | 186.71±44.69 | 13~83 |
| GLU (mmol/L) | 5.24±0.14 | 4.95±0.10 | 5.13±0.21 | 4.11~7.94 |
| BUN (mmol/L) | 2.48±0.16 | 3.36±0.28 | 3.23±0.20 | 2.5~9.6 |
| Cr (µmol/L) | 36.67±2.19 | 39.17±1.85 | 36.86±1.67 | 44~159 |
| TCHO (mmol/L) | 4.17±0.18 | 4.32±0.32 | 4.02±0.29 | 2.87~8.07 |
| CK (U/L) | 330.67±35.31 | 272.83±24.17 | 291.29±19.39 | 10~200 |
| **Blood routine^2^** |  |  |  |  |
| RBC (10^12^/L) | 5.95±0.17 | 6.18±0.11 | 5.92±0.16 | 4.29~8.5 |
| HGB (g/L) | 129.33±4.00 | 128.67±1.99 | 123.29±3.03 | 110~190 |
| HCT (%) | 39.03±1.11 | 39.05±0.76 | 37.20±0.88 | 35.0~49.0 |
| MCV (fL) | 65.65±0.56 | 63.23±0.40 | 62.89±1.05 | 60~76 |
| MCH (pg) | 21.75±0.11 | 20.87±0.19 | 20.81±0.22 | 22~27 |
| MCHC (g/L) | 331.50±2.36 | 329.67±2.23 | 331.43±2.76 | 300~380 |
| PLT (10^9^/L) | 368.83±37.52 | 391.33±20.93 | 375.00±41.27 | 117~490 |
| WBC (10^9^/L) | 14.43±1.47 | 14.95±1.56 | 14.90±0.52 | 5.0~17.0 |
| Gran# (10^9^/L) | 7.14±0.31 | 8.78±1.09 | 8.42±0.54 | 2.7~12.3 |
| Lymph# (10^9^/L) | 5.08±0.74 | 5.17±0.58 | 5.24±0.22 | 0.83~4.91 |
| Mon# (10^9^/L) | 0.64±0.06 | 0.61±0.05 | 0.87±0.06 | 0.1~1.97 |
| Eos% (%) | 5.38±1.28 | 4.24±1.21 | 3.40±0.50 | 0.5~10 |
| Gran% (%) | 53.90±2.88 | 58.32±1.97 | 56.20±2.07 | 52~75 |
| Lymph% (%) | 37.03±3.54 | 34.64±1.57 | 35.43±1.98 | 20.0~50.0 |

*^1^ ALT = Alanine aminotransferase; AST = Aspartate aminotransferase; TP = Total protein; ALB = Albumin; ALP = Alkaline phosphatase; GLU = Glucose; BUN = Blood urea nitrogen; Cr = Creatinine; TCHO = Total cholesterol; CK = Creatine kinase**.*

*^2^ RBC = Red blood cell count; HGB = Hemoglobin; HCT = Hematocrit; MCV = Mean corpuscular volume; MCH =* *Mean corpuscular hemoglobin;* *MCHC = Mean* *corpuscular hemoglobin concentration. PLT* *= Platelet count; WBC = White blood cell count; Gran = Granulocyte; Lymph = Lymphocyte; Mon = Monocyte; Eos = Eosinophil.*

**TABLE S2 |** The total list of fecal metabolites.

| **Number** | **Name** | **Formula** | **Molecular Weight** | **RT [min]** | **HMDB** | **PubChem** | **KEGG** |
| --- | --- | --- | --- | --- | --- | --- | --- |
| **1** | Adenosine monophosphate | C10 H14 N5 O7 P | 347.06274 | 1.32 | HMDB0000045 | 6083 | C00020 |
| **2** | L-Glutamic acid | C5 H9 N O4 | 147.05301 | 1.462 | HMDB0000148 | 33032 | C00025 |
| **3** | Succinic acid | C4 H6 O4 | 118.02759 | 1.865 | HMDB0000254 | 1110 | C00042 |
| **4** | Uridine diphosphate-N-acetylglucosamine | C17 H27 N3 O17 P2 | 112.02606 | 1.724 | HMDB0000290 | 9547196 | C00043 |
| **5** | L-Arginine | C6 H14 N4 O2 | 174.11161 | 1.735 | HMDB0000517 | 6322 | C00062 |
| **6** | L-Tyrosine | C9 H11 N O3 | 164.04735 | 1.428 | HMDB0000158 | 6057 | C00082 |
| **7** | L-Proline | C5 H9 N O2 | 115.06339 | 1.194 | HMDB0000162 | 145742 | C00148 |
| **8** | Niacinamide | C6 H6 N2 O | 122.04821 | 1.728 | HMDB0001406 | 936 | C00153 |
| **9** | PC(20:5(5Z,8Z,11Z,14Z,17Z)/P-18:1(11Z)) | C46 H80 N O7 P | 789.57138 | 6.525 | HMDB0008523 | 53479143 | C00157 |
| **10** | Citric acid | C6 H8 O7 | 192.02625 | 1.317 | HMDB0000094 | 311 | C00158 |
| **11** | L-Valine | C5 H11 N O2 | 117.07916 | 1.724 | HMDB0000883 | 6287 | C00183 |
| **12** | Adenosine | C10 H13 N5 O4 | 267.09649 | 1.904 | HMDB0000050 | 60961 | C00212 |
| **13** | Pyridoxal | C8 H9 N O3 | 167.05732 | 5.141 | HMDB0001545 | 1050 | C00250 |
| **14** | Nicotinic acid | C6 H5 N O2 | 123.03218 | 1.73 | HMDB0001488 | 938 | C00253 |
| **15** | Canavanine | C5 H12 N4 O3 | 176.09081 | 1.345 | HMDB0002706 | 439202 | C00308 |
| **16** | Deoxyadenosine monophosphate | C10 H14 N5 O6 P | 331.06862 | 4.783 | HMDB0000905 | 12599 | C00360 |
| **17** | 5-Thymidylic acid | C10 H15 N2 O8 P | 322.0563 | 1.731 | HMDB0001227 | 9700 | C00364 |
| **18** | Uric acid | C5 H4 N4 O3 | 168.02768 | 1.379 | HMDB0000289 | 1175 | C00366 |
| **19** | Xanthine | C5 H4 N4 O2 | 152.03339 | 1.394 | HMDB0000292 | 1188 | C00385 |
| **20** | Pyrimidine | C4 H4 N2 | 80.03795 | 1.367 | HMDB0003361 | 9260 | C00396 |
| **21** | Pipecolic acid | C6 H11 N O2 | 129.07905 | 1.725 | HMDB0000070 | 849 | C00408 |
| **22** | 5-Aminolevulinic acid | C5 H9 N O3 | 131.05705 | 1.64 | HMDB0001149 | 137 | C00430 |
| **23** | N-Carbamoylputrescine | C5 H13 N3 O | 131.10597 | 1.373 | HMDB0033458 | 502 | C00436 |
| **24** | Sulfoacetaldehyde | C2 H4 O4 S | 123.98197 | 1.312 | METPA0059 | NA | C00593 |
| **25** | Phenylacetaldehyde | C8 H8 O | 120.05765 | 1.568 | HMDB0006236 | 998 | C00601 |
| **26** | 4-Hydroxybenzaldehyde | C7 H6 O2 | 122.03693 | 1.428 | HMDB0011718 | 126 | C00633 |
| **27** | L-2-Amino-4-methylenepentanedioic acid | C6 H9 N O4 | 159.05306 | 1.729 | HMDB0029433 | 96407 | C00651 |
| **28** | Cholic acid | C24 H40 O5 | 408.28676 | 8.217 | HMDB0000619 | 221493 | C00695 |
| **29** | Tropine | C8 H15 N O | 141.11535 | 1.447 | METPA0074 | NA | C00729 |
| **30** | Serotonin | C10 H12 N2 O | 176.09496 | 2.426 | HMDB0000259 | 5202 | C00780 |
| **31** | Urocanic acid | C6 H6 N2 O2 | 138.04292 | 1.373 | HMDB0000301 | 736715 | C00785 |
| **32** | Biochanin A | C16 H12 O5 | 284.06835 | 5.1 | HMDB0002338 | 5280373 | C00814 |
| **33** | Phosphoglycolic acid | C2 H5 O6 P | 155.98335 | 17.985 | HMDB0000816 | 529 | C00988 |
| **34** | 4-Guanidinobutanoic acid | C5 H11 N3 O2 | 145.08515 | 1.376 | HMDB0003464 | 500 | C01035 |
| **35** | D-2-Hydroxyglutaric acid | C5 H8 O5 | 148.03607 | 1.433 | HMDB0000606 | 439391 | C01087 |
| **36** | 4-Hydroxyproline | C7 H11 N O4 | 173.06888 | 1.602 | HMDB0000725 | 5810 | C01157 |
| **37** | trans-Ferulic acid | C10 H10 O4 | 194.05697 | 5.158 | HMDB0000954 | 445858 | C01494 |
| **38** | Morphine | C17 H19 N O3 | 285.13603 | 7.16 | HMDB0014440 | 5288826 | C01516 |
| **39** | Linamarin | C10 H17 N O6 | 247.10596 | 1.738 | HMDB0033699 | 11128 | C01594 |
| **40** | Kynurenic acid | C10 H7 N O3 | 189.04229 | 3.918 | HMDB0000715 | 3845 | C01717 |
| **41** | Piperidine | C5 H11 N | 85.0896 | 1.39 | HMDB0034301 | 8082 | C01746 |
| **42** | Scopoletin | C10 H8 O4 | 192.04219 | 6.43 | HMDB0034344 | 5280460 | C01752 |
| **43** | Xanthosine | C10 H12 N4 O6 | 284.0754 | 2.067 | HMDB0000299 | 64959 | C01762 |
| **44** | Deoxyribose | C5 H10 O4 | 134.05674 | 1.521 | HMDB0003224 | 22833604 | C01801 |
| **45** | Haloperidol | C21 H23 Cl F N O2 | 375.13872 | 1.301 | HMDB0014645 | 3559 | C01814 |
| **46** | Rosmarinic acid | C18 H16 O8 | 360.08409 | 7.061 | HMDB0003572 | 5281792 | C01850 |
| **47** | Pyroglutamic acid | C5 H7 N O3 | 129.0422 | 1.739 | HMDB0000267 | 7405 | C01879 |
| **48** | Dethiobiotin | C10 H18 N2 O3 | 214.13182 | 1.414 | HMDB0003581 | 445027 | C01909 |
| **49** | Phenylglyoxylic acid | C8 H6 O3 | 150.03183 | 5.015 | HMDB0001587 | 11915 | C02137 |
| **50** | Glyceraldehyde | C3 H6 O3 | 90.03034 | 1.506 | HMDB0001051 | 751 | C02154 |
| **51** | D-Fructose | C6 H12 O6 | 90.03093 | 1.288 | HMDB0000660 | 439709 | C02336 |
| **52** | N-Acetylhistidine | C8 H11 N3 O3 | 197.08002 | 1.31 | HMDB0032055 | 273260 | C02997 |
| **53** | Tyramine glucuronide | C14 H19 N O7 | 313.11598 | 1.416 | HMDB0010328 | 193088 | C03033 |
| **54** | Pregnanediol-3-glucuronide | C27 H44 O8 | 496.30463 | 4.858 | HMDB0010318 | 123796 | C03033 |
| **55** | Phosphohydroxypyruvic acid | C3 H5 O7 P | 183.97818 | 17.964 | HMDB0001024 | 105 | C03232 |
| **56** | Quinolinic acid | C7 H5 N O4 | 167.02191 | 2.405 | HMDB0000232 | 1066 | C03722 |
| **57** | Malonic acid | C3 H4 O4 | 104.00973 | 1.409 | HMDB0000691 | 867 | C04025 |
| **58** | N1-(5-Phospho-a-D-ribosyl)-5,6-dimethylbenzimidazole | C14 H19 N2 O7 P | 358.09001 | 5.054 | HMDB0003882 | 444941 | C04778 |
| **59** | N-Acetylhistamine | C7 H11 N3 O | 153.09019 | 1.689 | HMDB0013253 | 69602 | C05135 |
| **60** | 2-Keto-6-acetamidocaproate | C8 H13 N O4 | 187.0845 | 1.37 | HMDB0012150 | 194080 | C05548 |
| **61** | Phosphonoacetate | C2 H5 O5 P | 139.98872 | 17.971 | HMDB0004110 | 546 | C05682 |
| **62** | D-Urobilin | C33 H40 N4 O6 | 588.29436 | 16.251 | HMDB0004161 | 6276321 | C05795 |
| **63** | Nicotinate D-ribonucleoside | C11 H13 N O6 | 255.07402 | 1.337 | HMDB0006809 | 161234 | C05841 |
| **64** | Benzophenone | C13 H10 O | 182.07309 | 10.89 | HMDB0032049 | 3102 | C06354 |
| **65** | Physostigmine | C15 H21 N3 O2 | 275.16328 | 4.039 | HMDB0015116 | 5983 | C06535 |
| **66** | Ampicillin | C16 H19 N3 O4 S | 349.11155 | 1.324 | HMDB0014559 | 6249 | C06574 |
| **67** | Carteolol | C16 H24 N2 O3 | 292.17826 | 6.328 | HMDB0014662 | 2583 | C06874 |
| **68** | Molindone | C16 H24 N2 O2 | 276.1836 | 2.193 | HMDB0015555 | 23897 | C07230 |
| **69** | Nafcillin | C21 H22 N2 O5 S | 414.12211 | 5.582 | HMDB0014745 | 8982 | C07250 |
| **70** | Olsalazine | C14 H10 N2 O6 | 302.05614 | 4.154 | HMDB0015380 | NA | C07323 |
| **71** | Pentobarbital | C11 H18 N2 O3 | 226.13165 | 1.835 | HMDB0014457 | 4737 | C07422 |
| **72** | Zaleplon | C17 H15 N5 O | 305.12925 | 8.262 | HMDB0015097 | 5719 | C07484 |
| **73** | Vigabatrin | C6 H11 N O2 | 129.07902 | 1.298 | HMDB0015212 | 5665 | C07500 |
| **74** | Pirenzepine | C19 H21 N5 O2 | 351.1708 | 8.674 | HMDB0014808 | 4848 | C07508 |
| **75** | N-Acetylarylamine | C8 H9 N O | 135.06846 | 1.427 | HMDB0001250 | 904 | C07565 |
| **76** | Ziprasidone | C21 H21 Cl N4 O S | 412.11266 | 7.42 | HMDB0014391 | 60854 | C07568 |
| **77** | Oxypurinol | C5 H4 N4 O2 | 152.03303 | 1.732 | HMDB0000786 | 4644 | C07599 |
| **78** | Nitrendipine | C18 H20 N2 O6 | 360.13063 | 1.352 | HMDB0015187 | 4507 | C07713 |
| **79** | Ibutilide | C20 H36 N2 O3 S | 384.24819 | 10.648 | HMDB0014453 | 60753 | C07753 |
| **80** | Cetirizine | C21 H25 Cl N2 O3 | 388.15911 | 2.095 | HMDB0005032 | 2678 | C07778 |
| **81** | Butabarbital | C10 H16 N2 O3 | 212.1161 | 1.781 | HMDB0014382 | 2479 | C07827 |
| **82** | Cinoxacin | C12 H10 N2 O5 | 262.05638 | 17.971 | HMDB0014965 | 2762 | C08052 |
| **83** | Jasmonic acid | C14 H18 O | 202.13402 | 1.381 | HMDB0015124 | 77991 | C11766 |
| **84** | 1H-Indole-3-carboxaldehyde | C9 H7 N O | 145.05165 | 4.771 | [HMDB0032797](http://www.hmdb.ca/metabolites/HMDB0032797) | [5281166](http://pubchem.ncbi.nlm.nih.gov/summary/summary.cgi?cid=5281166) | [C08491](http://www.genome.jp/dbget-bin/www_bget?C08491) |
| **85** | Yangonin | C15 H14 O4 | 258.08876 | 9.281 | HMDB0029737 | 10256 | C08493 |
| **86** | (S,E)-Zearalenone | C18 H22 O5 | 318.14395 | 4.879 | HMDB0034144 | 5281575 | C09980 |
| **87** | Americine | C31 H39 N5 O4 | 545.29587 | 2.655 | HMDB0031752 | 5375083 | C09981 |
| **88** | Picolinic acid | C6 H5 N O2 | 123.03083 | 2.402 | HMDB0034441 | 5855402 | C09996 |
| **89** | Proline betaine | C7 H13 N O2 | 143.09462 | 1.74 | HMDB0002243 | 1018 | C10164 |
| **90** | Physoperuvine | C8 H15 N O | 141.11539 | 6.013 | HMDB0004827 | 7016563 | C10172 |
| **91** | Adefovir Dipivoxil | C8 H12 N5 O4 P | 273.06028 | 1.282 | HMDB0030338 | 1.32E+08 | C10864 |
| **92** | Rivastigmine | C14 H22 N2 O2 | 250.16802 | 4.408 | HMDB0014856 | 60871 | C11277 |
| **93** | Tranexamic Acid | C8 H15 N O2 | 157.1103 | 1.359 | HMDB0031313 | 1623625 | C12288 |
| **94** | Prenyl caproate | C27 H40 O6 | 460.27925 | 10.558 | HMDB0014447 | NA | C12535 |
| **95** | Tetrahydrodeoxycorticosterone | C21 H34 O3 | 334.25035 | 12.684 | HMDB0032489 | 173534 | C13422 |
| **96** | Benzofuran | C8 H6 O | 118.04192 | 1.428 | HMDB0000879 | 101771 | C13713 |
| **97** | 2'-Deoxymugineic acid | C12 H20 N2 O7 | 304.12532 | 1.321 | HMDB0032929 | 9223 | C14512 |
| **98** | Aflatoxin G | C17 H12 O7 | 328.06059 | 5.755 | HMDB0033909 | 4641377 | C15485 |
| **99** | Maslinic acid | C30 H48 O4 | 472.35453 | 11.316 | HMDB0030474 | 14421 | C16755 |
| **100** | Astilbin | C21 H22 O11 | 450.11373 | 1.291 | HMDB0002392 | 73659 | C16939 |
| **101** | Ginsenoside Ro | C48 H76 O19 | 956.49636 | 8.863 | HMDB0033850 | 119258 | C17449 |
| **102** | (S)-3-Butyl-1(3H)-isobenzofuranone | C22 H34 O7 | 410.22757 | 1.924 | HMDB0034534 | NA | C17543 |
| **103** | Sulfamethazine | C12 H14 N4 O2 S | 278.08583 | 10.082 | HMDB0032064 | 61361 | C17854 |
| **104** | Ophthalmic acid | C11 H19 N3 O6 | 289.12664 | 1.365 | HMDB0015522 | 5327 | C19530 |
| **105** | Asparagoside A | C33 H54 O8 | 578.3813 | 11.465 | HMDB0005765 | 7018721 | C21016 |
| **106** | Ribothymidine | C10 H14 N2 O6 | 258.08489 | 1.791 | HMDB0030046 | 3390254 | NA |
| **107** | Cordeauxione | C14 H12 O7 | 292.05562 | 1.291 | HMDB0000884 | 445408 | NA |
| **108** | (±)-2-Methylthiazolidine | C4 H9 N S | 103.04594 | 1.431 | HMDB0034310 | NA | NA |
| **109** | N-Methyl-1H-indole-3-propanamide | C12 H14 N2 O | 202.1102 | 7.545 | HMDB0031682 | 32196 | NA |
| **110** | 3-Hydroxytetradecanedioic acid | C14 H26 O5 | 274.17772 | 8.011 | HMDB0032756 | 7661699 | NA |
| **111** | Cholesta-4,6-dien-3-one | C27 H42 O | 382.32268 | 10.977 | HMDB0000394 | 20848956 | NA |
| **112** | 8-Hydroxy-7-methylguanine | C6 H7 N5 O2 | 181.05974 | 1.943 | HMDB0002394 | 3034666 | NA |
| **113** | 8-Hydroxy-deoxyguanosine | C10 H13 N5 O5 | 283.09143 | 1.384 | HMDB0006037 | 308075 | NA |
| **114** | Acamprosate | C5 H11 N O4 S | 181.04 | 1.727 | HMDB0003333 | 73318 | NA |
| **115** | N-Acetylglutamine | C7 H12 N2 O4 | 188.07964 | 1.382 | HMDB0014797 | 71158 | NA |
| **116** | L-beta-aspartyl-L-aspartic acid | C8 H12 N2 O7 | 248.06441 | 1.301 | HMDB0006029 | 25561 | NA |
| **117** | Bosentan | C27 H29 N5 O6 S | 551.18901 | 13.929 | HMDB0011163 | 274178 | NA |
| **118** | Butalbital | C11 H16 N2 O3 | 224.11611 | 2.45 | HMDB0014699 | 104865 | NA |
| **119** | Chitotriose | C18 H35 N3 O13 | 501.21826 | 9.591 | HMDB0014386 | 2481 | NA |
| **120** | Cyclandelate | C17 H24 O3 | 276.17224 | 10.517 | HMDB0006578 | 121978 | NA |
| **121** | Deoxypyridinoline | C18 H28 N4 O7 | 412.19914 | 7.309 | HMDB0015586 | 2893 | NA |
| **122** | Dibutyl malate | C12 H22 O5 | 246.14616 | 7.695 | HMDB0000569 | 53477703 | NA |
| **123** | Diethyl malonate | C7 H12 O4 | 160.07258 | 1.532 | HMDB0031696 | 95385 | NA |
| **124** | Epinephrine sulfate | C9 H13 N O6 S | 263.04812 | 17.971 | [HMDB0001876](http://www.hmdb.ca/metabolites/HMDB0001876) | [3035453](http://pubchem.ncbi.nlm.nih.gov/summary/summary.cgi?cid=3035453) | NA |
| **125** | Epsilon-(gamma-Glutamyl)-lysine | C11 H21 N3 O5 | 275.14776 | 4.487 | HMDB0003869 | 7015685 | NA |
| **126** | Gabapentin | C9 H17 N O2 | 154.09925 | 6.386 | HMDB0005015 | 3446 | NA |
| **127** | Glipizide | C21 H27 N5 O4 S | 445.18006 | 1.322 | HMDB0015200 | 3478 | NA |
| **128** | Glucosylgalactosyl hydroxylysine | C18 H34 N2 O13 | 486.209 | 1.367 | HMDB0000585 | 122304 | NA |
| **129** | Glycitin | C22 H22 O10 | 446.12062 | 9.226 | HMDB0002219 | 12004532 | NA |
| **130** | Histidinyl-Aspartate | C10 H14 N4 O5 | 270.09518 | 1.263 | HMDB0028881 | 9860223 | NA |
| **131** | Homovanillic acid sulfate | C9 H10 O7 S | 262.014 | 4.363 | HMDB0011719 | 29981063 | NA |
| **132** | Hydroxyhexanoycarnitine | C13 H25 N O5 | 275.17297 | 5.357 | HMDB0013131 | 53481624 | NA |
| **133** | Isoleucyl-Cysteine | C9 H18 N2 O3 S | 234.10359 | 5.436 | HMDB0028904 | 3082857 | NA |
| **134** | Leucyl-Glutamine | C11 H21 N3 O4 | 259.15306 | 1.839 | HMDB0028927 | 4305457 | NA |
| **135** | L-leucyl-L-proline | C11 H20 N2 O3 | 228.14707 | 5.874 | HMDB11175 | 44369311 | NA |
| **136** | Lofexidine | C11 H12 Cl2 N2 O | 258.03458 | 1.966 | HMDB0015606 | 30668 | NA |
| **137** | Mesalazine | C7 H7 N O3 | 153.04143 | 9.135 | HMDB0014389 | 4075 | NA |
| **138** | Modafinil | C15 H15 N O2 S | 273.08436 | 1.741 | HMDB0014883 | 4236 | NA |
| **139** | Acetylglycine | C4 H7 N O3 | 117.04132 | 1.412 | HMDB0000532 | 10972 | NA |
| **140** | N-Acetylvaline | C7 H13 N O3 | 159.0895 | 1.326 | HMDB0011757 | 227752 | NA |
| **141** | N-Methoxy-1-vinyl-beta-carboline | C14 H12 N2 O | 224.09461 | 7.751 | HMDB0030379 | 639591 | NA |
| **142** | Tridecanoylglycine | C15 H29 N O3 | 271.21422 | 11.746 | HMDB0013317 | 45357453 | NA |
| **143** | N-Undecanoylglycine | C13 H25 N O3 | 243.1833 | 14.961 | HMDB0013286 | 454092 | NA |
| **144** | Oxyphenonium | C21 H34 N O3 | 348.25064 | 7.193 | HMDB0014364 | 5749 | NA |
| **145** | Paracetamol sulfate | C8 H9 N O5 S | 231.01929 | 2.017 | HMDB0059911 | 83939 | NA |
| **146** | Isoplumbagin | C11 H8 O3 | 188.04835 | 1.737 | HMDB0035291 | 375105 | NA |
| **147** | Propylparaben | C10 H12 O3 | 180.07855 | 10.531 | HMDB0032574 | 7175 | NA |
| **148** | Pyrrolidine | C4 H9 N | 71.07419 | 1.344 | HMDB0031641 | 31268 | NA |
| **149** | Rimexolone | C24 H34 O3 | 370.25044 | 7.216 | HMDB0015033 | 39507 | NA |
| **150** | Salicylamide | C7 H7 N O2 | 137.04765 | 1.727 | HMDB0015687 | 5147 | NA |
| **151** | Secobarbital | C12 H18 N2 O3 | 238.13156 | 2.552 | HMDB0014562 | 5193 | NA |
| **152** | Sitagliptin | C16 H15 F6 N5 O | 407.1187 | 1.295 | HMDB0015390 | 4369359 | NA |
| **153** | Trimethadione | C6 H9 N O3 | 143.0582 | 1.315 | HMDB0014491 | 5576 | NA |
| **154** | Verteporfin | C41 H42 N4 O8 | 718.30159 | 4.017 | HMDB0014603 | NA | NA |
| **155** | Vorinostat | C14 H20 N2 O3 | 264.14718 | 4.861 | HMDB0015568 | 5311 | NA |
| **156** | Aspartylphenylalanine | C13 H16 N2 O5 | 280.1057 | 4.974 | HMDB0000706 | 93078 | NA |

**TABLE S3 |** Results of analysis of the differential fecal metabolites at different time points.

| **Stage** | **No.** | **Compound name** | **Formula** | **Molecular**  **Weight** | **HMDB** | **KEGG** | **CON vs. TS** | | |  | **TS vs. TS+GA** | | |
| --- | --- | --- | --- | --- | --- | --- | --- | --- | --- | --- | --- | --- | --- |
|  |  |  |  |  |  |  | **Trend** | **VIP** | ***P*** |  | **Trend** | **VIP** | ***P*** |
| **BT1** |  |  |  |  |  |  |  |  |  |  |  |  |  |
|  | 1 | Deoxyadenosine monophosphate | C10H14N5O6P | 331.07 | HMDB0000905 | [C00360](http://www.genome.jp/dbget-bin/www_bget?C00360) | - | 0.43 | 0.79 |  | down | 2.23 | 0.013 |
|  | 2 | Ginsenoside Ro | C48H76O19 | 956.50 | HMDB0034534 | [C17543](http://www.genome.jp/dbget-bin/www_bget?C17543) | - | 1.26 | 0.44 |  | up | 2.20 | 0.014 |
|  | 3 | (S)-3-Butyl-1(3H)-isobenzofuranone | C12H14O2 | 190.24 | HMDB0032064 | [C17854](http://www.genome.jp/dbget-bin/www_bget?C17854) | - | 1.23 | 0.45 |  | down | 2.19 | 0.015 |
|  | 4 | Uridine diphosphate-N-acetylglucosamine | C17H27N3O17P2 | 607.35 | HMDB0000290 | [C00043](http://www.genome.jp/dbget-bin/www_bget?C00043) | - | 0.50 | 0.76 |  | down | 2.17 | 0.016 |
|  | 5 | Carteolol | C16H24N2O3 | 292.37 | HMDB0014662 | [C06874](http://www.genome.jp/dbget-bin/www_bget?C06874) | - | 1.28 | 0.43 |  | down | 2.02 | 0.029 |
|  | 6 | Phenylglyoxylic acid | C8H6O3 | 150.13 | HMDB0001587 | [C02137](http://www.genome.jp/dbget-bin/www_bget?C02137) | - | 0.62 | 0.71 |  | up | 1.98 | 0.033 |
| **AT1** |  |  |  |  |  |  |  |  |  |  |  |  |  |
|  | 1 | L-2-Amino-4-methylenepentanedioic acid | C6H9NO4 | 159.14 | [HMDB0029433](http://www.hmdb.ca/metabolites/HMDB0029433) | [C00651](http://www.genome.jp/dbget-bin/www_bget?C00651) | down | 2.43 | 0.050 |  | - | 0.14 | 0.87 |
|  | 2 | Phenylacetaldehyde | C8H8O | 120.15 | [HMDB0006236](http://www.hmdb.ca/metabolites/HMDB0006236) | [C00601](http://www.genome.jp/dbget-bin/www_bget?C00601) | - | 0.415 | 0.227 |  | up | 2.26 | 0.0006 |
|  | 3 | Benzophenone | C13H10O | 182.22 | [HMDB0032049](http://www.hmdb.ca/metabolites/HMDB0032049) | [C06354](http://www.genome.jp/dbget-bin/www_bget?C06354) | - | 0.753 | 0.579 |  | down | 2.04 | 0.004 |
|  | 4 | Phosphonoacetate | C2H5O5P | 140.03 | [HMDB0004110](http://www.hmdb.ca/metabolites/HMDB0004110) | [C05682](http://www.genome.jp/dbget-bin/www_bget?C05682) | - | 1.323 | 0.321 |  | down | 1.97 | 0.0061 |
|  | 5 | N-Acetylarylamine | C8H9NO | 135.16 | [HMDB0001250](http://www.hmdb.ca/metabolites/HMDB0001250) | [C07565](http://www.genome.jp/dbget-bin/www_bget?C07565) | - | 0.308 | 0.822 |  | up | 1.97 | 0.0062 |
|  | 6 | Benzofuran | C8H6O | 118.13 | [HMDB0032929](http://www.hmdb.ca/metabolites/HMDB0032929) | [C14512](http://www.genome.jp/dbget-bin/www_bget?C14512) | - | 0.305 | 0.824 |  | up | 1.97 | 0.0063 |
|  | 7 | Pirenzepine | C19H21N5O2 | 351.40 | [HMDB0014808](http://www.hmdb.ca/metabolites/HMDB0014808) | [C07508](http://www.genome.jp/dbget-bin/www_bget?C07508) | - | 0.194 | 0.887 |  | up | 1.94 | 0.0076 |
|  | 8 | L-Valine | C5H11NO2 | 117.15 | [HMDB0000883](http://www.hmdb.ca/metabolites/HMDB0000883) | [C00183](http://www.genome.jp/dbget-bin/www_bget?C00183) | - | 0.493 | 0.718 |  | up | 1.91 | 0.0089 |
|  | 9 | Phosphoglycolic acid | C2H5O6P | 156.03 | [HMDB0000816](http://www.hmdb.ca/metabolites/HMDB0000816) | [C00988](http://www.genome.jp/dbget-bin/www_bget?C00988) | - | 0.876 | 0.517 |  | down | 1.90 | 0.0095 |
|  | 10 | Deoxyadenosine monophosphate | C10H14N5O6P | 331.22 | [HMDB0000905](http://www.hmdb.ca/metabolites/HMDB0000905) | [C00360](http://www.genome.jp/dbget-bin/www_bget?C00360) | - | 0.589 | 0.666 |  | down | 1.88 | 0.0104 |
|  | 11 | Glyceraldehyde | C3H6O3 | 90.08 | [HMDB0001051](http://www.hmdb.ca/metabolites/HMDB0001051) | [C02154](http://www.genome.jp/dbget-bin/www_bget?C02154) | - | 1.596 | 0.226 |  | up | 1.72 | 0.0228 |
|  | 12 | Adefovir Dipivoxil | C20H32N5O8P | 501.47 | [HMDB0014856](http://www.hmdb.ca/metabolites/HMDB0014856) | [C11277](http://www.genome.jp/dbget-bin/www_bget?C11277) | - | 0.552 | 0.685 |  | up | 1.71 | 0.0246 |
|  | 13 | Phosphohydroxypyruvic acid | C3H5O7P | 184.04 | [HMDB0001024](http://www.hmdb.ca/metabolites/HMDB0001024) | [C03232](http://www.genome.jp/dbget-bin/www_bget?C03232) | - | 0.854 | 0.528 |  | down | 1.68 | 0.0272 |
|  | 14 | Cinoxacin | C12H10N2O5 | 262.22 | [HMDB0014965](http://www.hmdb.ca/metabolites/HMDB0014965) | [C08052](http://www.genome.jp/dbget-bin/www_bget?C08052) | - | 1.182 | 0.378 |  | down | 1.67 | 0.0290 |
|  | 15 | L-Tyrosine | C9H11NO3 | 181.19 | [HMDB0000158](http://www.hmdb.ca/metabolites/HMDB0000158) | [C00082](http://www.genome.jp/dbget-bin/www_bget?C00082) | - | 0.368 | 0.787 |  | up | 1.57 | 0.0422 |
|  | 16 | Olsalazine | C14H10N2O6 | 302.24 | [HMDB0015380](http://www.hmdb.ca/metabolites/HMDB0015380) | [C07323](http://www.genome.jp/dbget-bin/www_bget?C07323) | - | 1.738 | 0.184 |  | up | 1.56 | 0.0451 |
| **AT7** |  |  |  |  |  |  |  |  |  |  |  |  |  |
|  | 1 | Pregnanediol-3-glucuronide | C27H44O8 | 496.63 | [HMDB0010318](http://www.hmdb.ca/metabolites/HMDB0010318) | [C03033](http://www.genome.jp/dbget-bin/www_bget?C03033) | up | 2.73 | 0.027 |  | - | 0.424 | 0.584 |
|  | 2 | Americine | C31H39N5O4 | 545.67 | [HMDB0034441](http://www.hmdb.ca/metabolites/HMDB0034441) | [C09996](http://www.genome.jp/dbget-bin/www_bget?C09996) | up | 2.58 | 0.040 |  | down | 1.62 | 0.0184 |
|  | 3 | Vigabatrin | C6H11NO2 | 129.16 | [HMDB0015212](http://www.hmdb.ca/metabolites/HMDB0015212) | [C07500](http://www.genome.jp/dbget-bin/www_bget?C07500) | - | 1.003 | 0.467 |  | down | 1.77 | 0.0075 |
|  | 4 | Tetrahydrodeoxycorticosterone | C21H34O3 | 334.49 | [HMDB0000879](http://www.hmdb.ca/metabolites/HMDB0000879) | [C13713](http://www.genome.jp/dbget-bin/www_bget?C13713) | - | 0.809 | 0.559 |  | down | 1.56 | 0.0251 |
|  | 5 | Phosphoglycolic acid | C2H5O6P | 156.03 | [HMDB0000816](http://www.hmdb.ca/metabolites/HMDB0000816) | [C00988](http://www.genome.jp/dbget-bin/www_bget?C00988) | - | 0.745 | 0.591 |  | up | 1.55 | 0.0265 |
|  | 6 | Scopoletin | C10H8O4 | 192.17 | [HMDB0034344](http://www.hmdb.ca/metabolites/HMDB0034344) | [C01752](http://www.genome.jp/dbget-bin/www_bget?C01752) | - | 1.251 | 0.360 |  | up | 1.53 | 0.0282 |
|  | 7 | 2'-Deoxymugineic acid | C12H20N2O7 | 304.30 | [HMDB0033909](http://www.hmdb.ca/metabolites/HMDB0033909) | [C15485](http://www.genome.jp/dbget-bin/www_bget?C15485) | - | 0.444 | 0.750 |  | down | 1.51 | 0.0316 |
|  | 8 | Niacinamide | C6H6N2O | 122.12 | [HMDB0001406](http://www.hmdb.ca/metabolites/HMDB0001406) | [C00153](http://www.genome.jp/dbget-bin/www_bget?C00153) | - | 0.415 | 0.766 |  | down | 1.49 | 0.0342 |

**TABLE S4 |** The predominant potential fecal biomarkers based on the significant metabolic pathways.

| **Stage** | **Metabolism** | **Pathway name (*P* value)** | **Match metabolites** |
| --- | --- | --- | --- |
| **BT1**  **（TS vs. TS+GA）** | Glycan biosynthesis and metabolism | Glycosylphosphatidylinositol (GPI)-anchor biosynthesis (0.038855) | Uridine diphosphate-N-acetylglucosamine |
|  | Amino acid metabolism | Valine, leucine and isoleucine degradation (0.046359) | L-Valine |
|  | Amino acid metabolism | Valine, leucine and isoleucine biosynthesis (0.046359) | L-Valine |
|  | Metabolism of cofactors and vitamins | Pantothenate and CoA biosynthesis (0.046359) | L-Valine |
| **AT1**  **（CON vs. TS+GA）** | Lipid metabolism | Steroid hormone biosynthesis (0.0010051) | Tetrahydrodeoxycorticosterone |
|  | Amino acid metabolism | Phenylalanine metabolism (0.0057504) | Phenylacetaldehyde; L-Tyrosine |
|  | Metabolism of cofactors and vitamins | Ubiquinone and other terpenoid-quinone biosynthesis (0.0062924) | L-Tyrosine |
|  | Amino acid metabolism | Tyrosine metabolism (0.0062924) | L-Tyrosine |
|  | Amino acid metabolism | Phenylalanine, tyrosine and tryptophan biosynthesis (0.0062924) | L-Tyrosine |
|  | Genetic Information Processing; Translation | Aminoacyl-tRNA biosynthesis (0.0068787) | L-Arginine; L-Valine; L-Tyrosine; L-Proline; L-Glutamic acid |
|  | Amino acid metabolism | Valine, leucine and isoleucine degradation (0.0069715) | L-Valine |
|  | Amino acid metabolism | Valine, leucine and isoleucine biosynthesis (0.0069715) | L-Valine |
|  | Metabolism of cofactors and vitamins | Pantothenate and CoA biosynthesis (0.0069715) | L-Valine |
| **AT1**  **（TS vs. TS+GA）** | Amino acid metabolism | Phenylalanine metabolism (0.0088458) | Phenylacetaldehyde; L-Tyrosine |
|  | Genetic Information Processing; Translation | Aminoacyl-tRNA biosynthesis (0.0088882) | L-Arginine; L-Valine; L-Tyrosine; L-Proline; L-Glutamic acid |
|  | Metabolism of cofactors and vitamins | Ubiquinone and other terpenoid-quinone biosynthesis (0.0092432) | L-Tyrosine |
|  | Amino acid metabolism | Tyrosine metabolism (0.0092432) | L-Tyrosine |
|  | Amino acid metabolism | Phenylalanine, tyrosine and tryptophan biosynthesis (0.0092432) | L-Tyrosine |
|  | Amino acid metabolism | Valine, leucine and isoleucine degradation (0.011251) | L-Valine |
|  | Amino acid metabolism | Valine, leucine and isoleucine biosynthesis (0.011251) | L-Valine |
|  | Metabolism of cofactors and vitamins | Pantothenate and CoA biosynthesis (0.011251) | L-Valine |
|  | Lipid metabolism | Steroid hormone biosynthesis (0.029793) | Tetrahydrodeoxycorticosterone |
|  | Lipid metabolism | Glycerolipid metabolism (0.046858) | Glyceraldehyde |
|  | Carbohydrate metabolism | Fructose and mannose metabolism (0.047401) | D-Fructose; Glyceraldehyde |
| **AT7**  **（CON vs. TS+GA）** | Glycan biosynthesis and metabolism | Glycosylphosphatidylinositol (GPI)-anchor biosynthesis (0.023992) | Uridine diphosphate-N-acetylglucosamine |
|  | Amino acid metabolism | Tryptophan metabolism (0.03588) | Serotonin |
|  | Carbohydrate metabolism | Purine metabolism (0.036288) | Xanthine; Adenosine monophosphate; Adenosine; Deoxyadenosine monophosphate; Xanthosine; Uric acid |
| **AT7**  **（TS vs. TS+GA）** | Carbohydrate metabolism | Glyoxylate and dicarboxylate metabolism (0.045367) | Citric acid; Phosphoglycolic acid; L-Glutamic acid |

**TABLE S5 |** The total list of serum metabolites.

| **Number** | **Compound name** | **Formula** | **Molecular Weight** | **RT [min]** | **HMDB** | **PubChem** | **KEGG** |
| --- | --- | --- | --- | --- | --- | --- | --- |
| **1** | Phosphoric acid | H3 O4 P | 97.9661 | 1.94 | HMDB0002142 | 57424078 | C00009 |
| **2** | Pyruvic acid | C3 H4 O3 | 88.01477 | 1.421 | HMDB0000243 | 1060 | C00022 |
| **3** | L-Glutamic acid | C5 H9 N O4 | 147.05286 | 1.457 | HMDB0000148 | 33032 | C00025 |
| **4** | L-Lysine | C6 H14 N2 O2 | 146.10526 | 1.122 | HMDB0000182 | 5962 | C00047 |
| **5** | L-Arginine | C6 H14 N4 O2 | 174.11128 | 1.186 | HMDB0000517 | 6322 | C00062 |
| **6** | L-Glutamine | C5 H10 N2 O3 | 146.06846 | 1.281 | HMDB0000641 | 5961 | C00064 |
| **7** | L-Tryptophan | C11 H12 N2 O2 | 204.08915 | 4.591 | HMDB0000929 | 6305 | C00078 |
| **8** | L-Phenylalanine | C9 H11 N O2 | 165.07793 | 3.188 | HMDB0000159 | 6140 | C00079 |
| **9** | 2-Aminobenzoic acid | C7 H7 N O2 | 137.04759 | 1.291 | HMDB0001123 | 227 | C00108 |
| **10** | Biotin | C10 H16 N2 O3 S | 222.1098 | 4.874 | HMDB0000030 | 171548 | C00120 |
| **11** | L-Histidine | C6 H9 N3 O2 | 155.0692 | 1.128 | HMDB0000177 | 6274 | C00135 |
| **12** | L-Proline | C5 H9 N O2 | 115.06332 | 1.299 | HMDB0000162 | 145742 | C00148 |
| **13** | Niacinamide | C6 H6 N2 O | 122.0478 | 1.73 | HMDB0001406 | 936 | C00153 |
| **14** | Citric acid | C6 H8 O7 | 192.02604 | 1.752 | HMDB0000094 | 311 | C00158 |
| **15** | L-Valine | C5 H11 N O2 | 117.07887 | 1.291 | HMDB0000883 | 6287 | C00183 |
| **16** | Arachidonic acid | C20 H32 O2 | 304.23915 | 14.146 | HMDB0001043 | 444899 | C00219 |
| **17** | Taurine | C2 H7 N O3 S | 125.01344 | 1.253 | HMDB0000251 | 1123 | C00245 |
| **18** | L-Lactic acid | C3 H6 O3 | 90.03034 | 1.732 | HMDB0000190 | 61503 | C00256 |
| **19** | Hypoxanthine | C5 H4 N4 O | 136.03813 | 1.734 | HMDB0000157 | 790 | C00262 |
| **20** | Inosine | C10 H12 N4 O5 | 115.06204 | 1.834 | HMDB0000195 | 6021 | C00294 |
| **21** | Creatine | C4 H9 N3 O2 | 131.06924 | 1.288 | HMDB0000064 | 586 | C00300 |
| **22** | Spermidine | C7 H19 N3 | 145.15758 | 1.111 | HMDB0001257 | 1102 | C00315 |
| **23** | L-Carnitine | C7 H15 N O3 | 161.10477 | 1.304 | HMDB0000062 | 2724480 | C00318 |
| **24** | Citrulline | C6 H13 N3 O3 | 175.09495 | 1.273 | HMDB0000904 | 9750 | C00327 |
| **25** | Uric acid | C5 H4 N4 O3 | 168.02745 | 1.724 | HMDB0000289 | 1175 | C00366 |
| **26** | Carnosine | C9 H14 N4 O3 | 113.0532 | 1.111 | HMDB0000033 | 439224 | C00386 |
| **27** | L-Isoleucine | C6 H13 N O2 | 131.09433 | 2.177 | HMDB0000172 | 6306 | C00407 |
| **28** | Pipecolic acid | C6 H11 N O2 | 88.05248 | 1.713 | HMDB0000070 | 849 | C00408 |
| **29** | N-Acetylornithine | C7 H14 N2 O3 | 174.09991 | 1.314 | HMDB0003357 | 439232 | C00437 |
| **30** | Indole | C8 H7 N | 117.05754 | 4.592 | HMDB0000738 | 798 | C00463 |
| **31** | Glutaric acid | C5 H8 O4 | 132.04111 | 3.701 | HMDB0000661 | 743 | C00489 |
| **32** | Benzoic acid | C7 H6 O2 | 122.03544 | 11.576 | HMDB0001870 | 243 | C00539 |
| **33** | Cyclohexylamine | C6 H13 N | 99.10502 | 2.948 | HMDB0031404 | 7965 | C00571 |
| **34** | Phosphorylcholine | C5 H15 N O4 P | 183.06691 | 11.679 | HMDB0001565 | 8691 | C00588 |
| **35** | 4-Hydroxybenzaldehyde | C7 H6 O2 | 122.03576 | 4.76 | HMDB0011718 | 126 | C00633 |
| **36** | Prostaglandin F2a | C20 H34 O5 | 354.24028 | 9.354 | HMDB0001139 | 5283078 | C00639 |
| **37** | Cholic acid | C24 H40 O5 | 408.28656 | 8.941 | HMDB0000619 | 221493 | C00695 |
| **38** | Oleic acid | C18 H34 O2 | 282.25465 | 12.776 | HMDB0000207 | 445639 | C00712 |
| **39** | Salicylic acid | C7 H6 O3 | 138.03052 | 5.124 | HMDB0001895 | 338 | C00805 |
| **40** | D-Glutamine | C5 H10 N2 O3 | 146.06882 | 1.972 | HMDB0003423 | 145815 | C00819 |
| **41** | Pantothenic acid | C9 H17 N O5 | 219.1101 | 3.652 | HMDB0000210 | 6613 | C00864 |
| **42** | 4-Nitrophenol | C6 H5 N O3 | 139.02581 | 7.972 | HMDB0001232 | 980 | C00870 |
| **43** | Dihydrothymine | C5 H8 N2 O2 | 128.05834 | 1.3 | HMDB0000079 | 93556 | C00906 |
| **44** | Indoleacetic acid | C10 H9 N O2 | 175.06287 | 7.658 | HMDB0000197 | 802 | C00954 |
| **45** | Prostaglandin B1 | C20 H32 O4 | 336.22904 | 12.122 | HMDB0002982 | 5280388 | C00959 |
| **46** | D-2-Hydroxyglutaric acid | C5 H8 O5 | 148.036 | 1.736 | HMDB0000606 | 439391 | C01087 |
| **47** | 3-Hydroxybutyric acid | C4 H8 O3 | 104.04607 | 2.044 | HMDB0000357 | 441 | C01089 |
| **48** | Trimethylamine N-oxide | C3 H9 N O | 75.06882 | 1.286 | HMDB0000925 | 1145 | C01104 |
| **49** | 4-Hydroxyproline | C5 H9 N O3 | 131.05827 | 1.286 | HMDB0000725 | 5810 | C01157 |
| **50** | Phenyllactic acid | C9 H10 O3 | 166.06194 | 6.657 | HMDB0000779 | 3848 | C01479 |
| **51** | Capric acid | C10 H20 O2 | 172.14528 | 10.558 | HMDB0000511 | 2969 | C01571 |
| **52** | Caproic acid | C6 H12 O2 | 116.08245 | 7.105 | HMDB0000535 | 8892 | C01585 |
| **53** | Hippuric acid | C9 H9 N O3 | 179.0573 | 5.499 | HMDB0000714 | 464 | C01586 |
| **54** | Linoleic acid | C18 H32 O2 | 280.24028 | 12.087 | HMDB0000673 | 5280450 | C01595 |
| **55** | Melatonin | C13 H16 N2 O2 | 232.12045 | 7.356 | HMDB0001389 | 896 | C01598 |
| **56** | Phthalic acid | C8 H6 O4 | 166.02606 | 11.573 | HMDB0002107 | 1017 | C01606 |
| **57** | Kynurenic acid | C10 H7 N O3 | 189.042 | 4.833 | HMDB0000715 | 3845 | C01717 |
| **58** | Piperidine | C5 H11 N | 85.08942 | 2.178 | HMDB0034301 | 8082 | C01746 |
| **59** | But-2-enoic acid | C4 H6 O2 | 86.03686 | 2.045 | HMDB0010720 | 637090 | C01771 |
| **60** | 2-Hydroxycinnamic acid | C9 H8 O3 | 164.047 | 3.679 | HMDB0002641 | 637540 | C01772 |
| **61** | Pyroglutamic acid | C5 H7 N O3 | 129.04203 | 1.739 | HMDB0000267 | 7405 | C01879 |
| **62** | Indolelactic acid | C11 H11 N O3 | 205.0731 | 6.915 | HMDB0000671 | 92904 | C02043 |
| **63** | Glyceraldehyde | C3 H6 O3 | 90.03032 | 1.832 | HMDB0001051 | 751 | C02154 |
| **64** | Glutaconic acid | C5 H6 O4 | 130.02544 | 3.237 | HMDB0000620 | 5280498 | C02214 |
| **65** | trans-Aconitic acid | C6 H6 O6 | 174.0154 | 1.749 | HMDB0000958 | 444212 | C02341 |
| **66** | Chenodeoxycholic acid | C24 H40 O4 | 392.29161 | 10.33 | HMDB0000518 | 10133 | C02528 |
| **67** | Ureidopropionic acid | C4 H8 N2 O3 | 132.05312 | 1.401 | HMDB0000026 | 111 | C02642 |
| **68** | 4-Coumaryl alcohol | C9 H6 O3 | 180.04163 | 12.95 | HMDB0003654 | 54682930 | C02646 |
| **69** | D-Glucurono-6,3-lactone | C6 H8 O6 | 176.03111 | 8.125 | HMDB0006355 | 439782 | C02670 |
| **70** | Dodecanedioic acid | C12 H22 O4 | 230.15112 | 9.585 | HMDB0000623 | 12736 | C02678 |
| **71** | Dodecanoic acid | C12 H24 O2 | 200.17675 | 11.419 | HMDB0000638 | 3893 | C02679 |
| **72** | 2,3-Dihydroxytoluene | C7 H8 O2 | 124.05104 | 5.851 | METPA0335 | NA | C02923 |
| **73** | N-Acetylhistidine | C8 H11 N3 O3 | 197.07966 | 1.113 | HMDB0032055 | 273260 | C02997 |
| **74** | Hydroxyphenyllactic acid | C9 H10 O4 | 182.05699 | 4.562 | HMDB0000755 | 9378 | C03672 |
| **75** | Alpha-N-Phenylacetyl-L-glutamine | C13 H16 N2 O4 | 264.11047 | 5.517 | HMDB0006344 | 92258 | C04148 |
| **76** | LysoPC(16:0) | C24 H50 N O7 P | 495.32974 | 12.499 | HMDB0010382 | 460602 | C04230 |
| **77** | Deoxycholic acid | C24 H40 O4 | 392.29203 | 11.167 | HMDB0000626 | 222528 | C04483 |
| **78** | 13-L-Hydroperoxylinoleic acid | C18 H32 O4 | 312.2295 | 11.636 | HMDB0003871 | 6437847 | C04717 |
| **79** | Taurochenodesoxycholic acid | C26 H45 N O6 S | 499.29625 | 9.883 | HMDB0000951 | 387316 | C05465 |
| **80** | Homovanillic acid | C9 H10 O4 | 182.05706 | 4.76 | HMDB0000118 | 1738 | C05582 |
| **81** | 3-Hydroxyphenylacetic acid | C8 H8 O3 | 152.04623 | 5.385 | HMDB0000440 | 12122 | C05593 |
| **82** | Phenylacetylglycine | C10 H11 N O3 | 193.07321 | 6.045 | HMDB0000821 | 68144 | C05598 |
| **83** | 5-Hydroxyindoleacetic acid | C10 H9 N O3 | 191.0577 | 3.155 | HMDB0000763 | 1826 | C05635 |
| **84** | 4,6-Dihydroxyquinoline | C9 H7 N O2 | 161.04718 | 6.597 | HMDB0004077 | 440738 | C05639 |
| **85** | Pantothenol | C9 H19 N O4 | 205.1309 | 3.659 | HMDB0004231 | 4678 | C05944 |
| **86** | Thromboxane B2 | C20 H34 O6 | 370.23489 | 9.289 | HMDB0003252 | 5283137 | C05963 |
| **87** | Adipic acid | C6 H10 O4 | 146.05682 | 4.356 | HMDB0000448 | 196 | C06104 |
| **88** | Isoquinoline | C9 H7 N | 129.05743 | 10.278 | HMDB0034244 | 8405 | C06323 |
| **89** | Terephthalic acid | C8 H6 O4 | 166.02556 | 5.45 | HMDB0002428 | 7489 | C06337 |
| **90** | Caprylic acid | C8 H16 O2 | 144.1139 | 9.298 | HMDB0000482 | 379 | C06423 |
| **91** | Alpha-Linolenic acid | C18 H30 O2 | 278.22342 | 11.538 | HMDB0001388 | 5280934 | C06427 |
| **92** | Acetaminophen | C8 H9 N O2 | 151.0628 | 1.736 | HMDB0001859 | 1983 | C06804 |
| **93** | Phenylacetic acid | C8 H8 O2 | 136.05124 | 5.324 | HMDB0000209 | 999 | C07086 |
| **94** | Metronidazole | C6 H9 N3 O3 | 171.06389 | 4.048 | HMDB0015052 | 4173 | C07203 |
| **95** | Methacholine | C8 H17 N O2 | 159.12555 | 1.353 | HMDB0015654 | 1993 | C07471 |
| **96** | N-Acetylarylamine | C8 H9 N O | 135.06816 | 7.462 | HMDB0001250 | 904 | C07565 |
| **97** | Miconazole | C18 H14 Cl4 N2 O | 413.98068 | 5.369 | HMDB0015242 | 68553 | C08070 |
| **98** | Palmitoleic acid | C16 H30 O2 | 254.22352 | 11.694 | HMDB0003229 | 5312427 | C08362 |
| **99** | Indole-3-carboxaldehyde | C9 H7 N O | 145.05235 | 4.593 | HMDB0029737 | 10256 | C08493 |
| **100** | Methylsuccinic acid | C5 H8 O4 | 132.04107 | 2.721 | HMDB0001844 | 10349 | C08645 |
| **101** | Lenticin | C14 H18 N2 O2 | 246.13623 | 5.08 | HMDB0061115 | 442106 | C09213 |
| **102** | trans-Cinnamic acid | C9 H8 O2 | 148.05196 | 3.188 | HMDB0000930 | 444539 | C10438 |
| **103** | Syringic acid | C9 H10 O5 | 198.05168 | 5.366 | HMDB0002085 | 10742 | C10833 |
| **104** | 3-Indolebutyric acid | C12 H13 N O2 | 203.09379 | 10.278 | HMDB0002096 | 8617 | C11284 |
| **105** | p-Hydroxymandelic acid | C8 H8 O4 | 168.04123 | 2.291 | HMDB0000822 | 7721 | C11527 |
| **106** | Phytosphingosine | C18 H39 N O3 | 317.29154 | 10.246 | HMDB0004610 | 122121 | C12144 |
| **107** | m-Coumaric acid | C9 H8 O3 | 164.04602 | 6.381 | HMDB0001713 | 637541 | C12621 |
| **108** | 3-Cresotinic acid | C8 H8 O3 | 152.04623 | 6.262 | HMDB0002390 | 6738 | C14088 |
| **109** | Dibutyl phthalate | C16 H22 O4 | 278.15108 | 11.573 | HMDB0033244 | 3026 | C14214 |
| **110** | (±)-2-(1-Methylpropyl)-4,6-dinitrophenol | C10 H12 N2 O5 | 240.07403 | 11.702 | HMDB0032559 | 6950 | C14302 |
| **111** | 5-KETE | C20 H30 O3 | 318.21871 | 12.653 | HMDB0010217 | 5283159 | C14732 |
| **112** | 20-Hydroxyeicosatetraenoic acid | C20 H32 O3 | 320.23421 | 11.506 | HMDB0005998 | 5283157 | C14748 |
| **113** | 11,12-Epoxyeicosatrienoic acid | C20 H32 O3 | 320.23441 | 12.09 | HMDB0004673 | 5353269 | C14770 |
| **114** | 16(R)-HETE | C20 H32 O3 | 302.22335 | 12.096 | HMDB0004680 | 9548884 | C14778 |
| **115** | 9,10-DHOME | C18 H34 O4 | 314.24524 | 10.957 | HMDB0004704 | 9966640 | C14828 |
| **116** | Phenol | C6 H6 O | 94.04037 | 5.06 | HMDB0000228 | 996 | C15584 |
| **117** | gamma-Asarone | C12 H16 O3 | 208.10933 | 8.576 | HMDB0029872 | 636750 | C17821 |
| **118** | 16-Hydroxy hexadecanoic acid | C16 H32 O3 | 272.23468 | 11.694 | HMDB0006294 | 7058075 | C18218 |
| **119** | Hexadecanedioic acid | C16 H30 O4 | 286.21381 | 11.176 | HMDB0000672 | 10459 | C19615 |
| **120** | Oleamide | C18 H35 N O | 281.27056 | 14.543 | HMDB0002117 | 5283387 | C19670 |
| **121** | 6-Methylquinoline | C10 H9 N | 143.07315 | 4.592 | HMDB0033115 | 7059 | NA |
| **122** | Indole-3-methyl acetate | C11 H11 N O2 | 189.07823 | 9.633 | HMDB0029738 | 74706 | NA |
| **123** | Indole-3-propionic acid | C11 H11 N O2 | 189.07828 | 8.538 | HMDB0002302 | 3744 | NA |
| **124** | gamma-Glutamylleucine | C11 H20 N2 O5 | 260.13656 | 4.849 | HMDB0011171 | 4524287 | NA |
| **125** | Indoleacrylic acid | C11 H9 N O2 | 187.06274 | 4.592 | HMDB0000734 | 15030923 | NA |
| **126** | Tetradecanedioic acid | C14 H26 O4 | 258.18251 | 10.536 | HMDB0000872 | 13185 | NA |
| **127** | Palmitic amide | C16 H33 N O | 255.25511 | 14.399 | HMDB0012273 | 69421 | NA |
| **128** | 9-HODE | C18 H32 O3 | 296.2345 | 11.783 | HMDB0010223 | 5282945 | NA |
| **129** | Octadecanedioic acid | C18 H34 O4 | 314.24505 | 12.118 | HMDB0000782 | 70095 | NA |
| **130** | Linoleoyl ethanolamide | C20 H37 N O2 | 323.28112 | 12.986 | HMDB0012252 | 5283446 | NA |
| **131** | LysoPE(16:0/0:0) | C21 H44 N O7 P | 453.28406 | 12.072 | HMDB0011503 | 9547069 | NA |
| **132** | LysoPE(18:2(9Z,12Z)/0:0) | C23 H43 N O7 P | 477.28471 | 11.44 | HMDB0011507 | 52925130 | NA |
| **133** | Alpha-Hydroxyisobutyric acid | C4 H8 O3 | 104.04606 | 2.337 | HMDB0000729 | 11671 | NA |
| **134** | 2-Hydroxy-2-methylbutyric acid | C5 H10 O3 | 118.06178 | 3.998 | HMDB0001987 | 95433 | NA |
| **135** | 3-Hydroxyvaleric acid | C5 H10 O3 | 118.06177 | 4.477 | HMDB0000531 | 107802 | NA |
| **136** | (+/-)-2-Hydroxy-4-(methylthio)butanoic acid | C5 H10 O3 S | 150.0341 | 4.499 | HMDB37115 | 11427 | NA |
| **137** | 2-Piperidinone | C5 H9 N O | 99.06856 | 3.232 | HMDB0011749 | 12665 | NA |
| **138** | N-Acetyl-L-alanine | C5 H9 N O3 | 131.05709 | 1.978 | HMDB0000766 | 88064 | NA |
| **139** | N-Propionylmethionine | C6 H11 N O2 | 129.07878 | 1.356 | HMDB0094704 | 21117855 | NA |
| **140** | 2-Hydroxycaproic acid | C6 H12 O3 | 132.07744 | 6.019 | HMDB0001624 | 99824 | NA |
| **141** | Leucinic acid | C6 H12 O3 | 132.07744 | 6.121 | HMDB0000665 | 92779 | NA |
| **142** | 2,4-Dihydroxybenzoic acid | C7 H6 O4 | 154.02551 | 6.091 | HMDB0029666 | 1491 | NA |
| **143** | Glycyl-Isoleucine | C8 H16 N2 O3 | 188.1156 | 2.347 | HMDB0028844 | 88079 | NA |
| **144** | Indoxyl sulfate | C8 H7 N O4 S | 213.00876 | 5.597 | HMDB0000682 | 10258 | NA |
| **145** | 4-O-Methylgallic acid | C8 H8 O5 | 184.03624 | 4.604 | HMDB0013198 | 78016 | NA |
| **146** | 4-Methoxyphenylacetic acid | C9 H10 O3 | 166.0619 | 5.667 | HMDB0002072 | 7690 | NA |
| **147** | N-acetyl-5-aminosalicylic acid | C9 H9 N O4 | 195.0523 | 7.534 | HMDB0060602 | 65512 | NA |

**TABLE S6 |** Results of analysis of the differential serum metabolites.

| **Stage** | **No.** | **Compound name** | **Formula** | **Molecular Weight** | **HMDB** | **KEGG** | **CON vs. TS** | | |  | **TS vs. TS+GA** | | |
| --- | --- | --- | --- | --- | --- | --- | --- | --- | --- | --- | --- | --- | --- |
|  |  |  |  |  |  |  | **Trend** | **VIP** | ***P*** |  | **Trend** | **VIP** | ***P*** |
| **BT1** |  |  |  |  |  |  |  |  |  |  |  |  |  |
|  | 1 | 2,3-Dihydroxytoluene | C7H8O2 | 124.1372 | [METPA0335](http://www.hmdb.ca/metabolites/METPA0335) | [C02923](http://www.genome.jp/dbget-bin/www_bget?C02923) | down | 2.287 | 0.045 |  | - | 0.255 | 0.784 |
|  | 2 | 4-O-Methylgallic acid | C8H8O5 | 184.1461 | [HMDB0013198](http://www.hmdb.ca/metabolites/HMDB0013198) | -- | - | 0.267 | 0.833 |  | down | 2.9741 | 9.61E-10 |
|  | 3 | Miconazole | C18H14Cl4N2O | 416.129 | [HMDB0015242](http://www.hmdb.ca/metabolites/HMDB0015242) | [C08070](http://www.genome.jp/dbget-bin/www_bget?C08070) | - | 0.981 | 0.431 |  | down | 2.9307 | 3.77E-08 |
|  | 4 | Syringic acid | C9H10O5 | 198.174 | [HMDB0002085](http://www.hmdb.ca/metabolites/HMDB0002085) | C10833 | - | 0.175 | 0.890 |  | down | 2.5987 | 0.0002 |
|  | 5 | L-Carnitine | C7H16NO3 | 162.2068 | [HMDB0000062](http://www.hmdb.ca/metabolites/HMDB0000062) | [C00318](http://www.genome.jp/dbget-bin/www_bget?C00318) | - | 0.685 | 0.585 |  | down | 2.298 | 0.0025 |
|  | 6 | Phytosphingosine | C18H39NO3 | 317.5072 | [HMDB0004610](http://www.hmdb.ca/metabolites/HMDB0004610) | [C12144](http://www.genome.jp/dbget-bin/www_bget?C12144) | - | 1.220 | 0.323 |  | up | 2.1592 | 0.006 |
|  | 7 | Glutaconic acid | C5H6O4 | 130.0987 | [HMDB0000620](http://www.hmdb.ca/metabolites/HMDB0000620) | [C02214](http://www.genome.jp/dbget-bin/www_bget?C02214) | - | 1.504 | 0.216 |  | down | 1.8984 | 0.0213 |
|  | 8 | Pyroglutamic acid | C5H7NO3 | 129.114 | [HMDB0000267](http://www.hmdb.ca/metabolites/HMDB0000267) | [C01879](http://www.genome.jp/dbget-bin/www_bget?C01879) | - | 0.396 | 0.754 |  | up | 1.8516 | 0.0258 |
|  | 9 | 4,6-Dihydroxyquinoline | C9H7NO2 | 161.1574 | [HMDB0004077](http://www.hmdb.ca/metabolites/HMDB0004077) | [C05639](http://www.genome.jp/dbget-bin/www_bget?C05639) | - | 1.059 | 0.394 |  | down | 1.8434 | 0.0267 |
|  | 10 | Hypoxanthine | C5H4N4O | 136.1115 | [HMDB0000157](http://www.hmdb.ca/metabolites/HMDB0000157) | [C00262](http://www.genome.jp/dbget-bin/www_bget?C00262) | - | 2.195 | 0.057 |  | up | 1.8431 | 0.0267 |
|  | 11 | 4-Coumaryl alcohol | C9H10O2 | 150.1745 | [HMDB0003654](http://www.hmdb.ca/metabolites/HMDB0003654) | [C02646](http://www.genome.jp/dbget-bin/www_bget?C02646) | - | 1.227 | 0.320 |  | down | 1.7674 | 0.0356 |
|  | 12 | Methacholine | C8H18NO2 | 160.234 | [HMDB0015654](http://www.hmdb.ca/metabolites/HMDB0015654) | [C07471](http://www.genome.jp/dbget-bin/www_bget?C07471) | - | 2.026 | 0.084 |  | up | 1.7333 | 0.0402 |
|  | 13 | Dibutyl phthalate | C16H22O4 | 278.3435 | [HMDB0033244](http://www.hmdb.ca/metabolites/HMDB0033244) | [C14214](http://www.genome.jp/dbget-bin/www_bget?C14214) | - | 0.395 | 0.754 |  | down | 1.689 | 0.0468 |
| **AT1** |  |  |  |  |  |  |  |  |  |  |  |  |  |
|  | 1 | 3-Hydroxybutyric acid | C4H8O3 | 104.1045 | [HMDB0000357](http://www.hmdb.ca/metabolites/HMDB0000357) | [C01089](http://www.genome.jp/dbget-bin/www_bget?C01089) | up | 2.1728 | 4.85E-06 |  | - | 1.2885 | 0.11233 |
|  | 2 | Dibutyl phthalate | C16H22O4 | 278.3435 | [HMDB0033244](http://www.hmdb.ca/metabolites/HMDB0033244) | [C14214](http://www.genome.jp/dbget-bin/www_bget?C14214) | down | 2.1393 | 1.43E-05 |  | - | 0.79719 | 0.34422 |
|  | 3 | Phthalic acid | [C8H6O4](https://pubchem.ncbi.nlm.nih.gov/#query=C8H6O4) | 166.1308 | [HMDB0002107](http://www.hmdb.ca/metabolites/HMDB0002107) | [C01606](http://www.genome.jp/dbget-bin/www_bget?C01606) | down | 2.1299 | 1.87E-05 |  | - | 0.66859 | 0.4306 |
|  | 4 | But-2-enoic acid | [C4H6O2](https://pubchem.ncbi.nlm.nih.gov/#query=C4H6O2) | 86.0892 | [HMDB0010720](http://www.hmdb.ca/metabolites/HMDB0010720) | [C01771](http://www.genome.jp/dbget-bin/www_bget?C01771) | up | 2.0096 | 0.000227 |  | - | 0.61503 | 0.46947 |
|  | 5 | Benzoic acid | [C7H6O2](https://pubchem.ncbi.nlm.nih.gov/#query=C7H6O2) | 122.123 | [HMDB0001870](http://www.hmdb.ca/metabolites/HMDB0001870) | [C00539](http://www.genome.jp/dbget-bin/www_bget?C00539) | down | 2.0066 | 0.000238 |  | - | 0.81156 | 0.3352 |
|  | 6 | 4-Coumaryl alcohol | [C9H6O3](https://pubchem.ncbi.nlm.nih.gov/#query=C9H6O3) | 150.1745 | [HMDB0003654](http://www.hmdb.ca/metabolites/HMDB0003654) | [C02646](http://www.genome.jp/dbget-bin/www_bget?C02646) | down | 1.9868 | 0.000322 |  | - | 0.2809 | 0.74358 |
|  | 7 | Spermidine | [C7H19N3](https://pubchem.ncbi.nlm.nih.gov/#query=C7H19N3) | 145.2459 | [HMDB0001257](http://www.hmdb.ca/metabolites/HMDB0001257) | [C00315](http://www.genome.jp/dbget-bin/www_bget?C00315) | up | 1.9062 | 0.000929 |  | - | 0.073528 | 0.93193 |
|  | 8 | Phenyllactic acid | [C9H10O3](https://pubchem.ncbi.nlm.nih.gov/#query=C9H10O3) | 166.1739 | [HMDB0000779](http://www.hmdb.ca/metabolites/HMDB0000779) | [C01479](http://www.genome.jp/dbget-bin/www_bget?C01479) | down | 1.8351 | 0.001986 |  | - | 1.3483 | 0.094509 |
|  | 9 | 2,4-Dihydroxybenzoic acid | C7H6O4 | 154.121 | [HMDB0029666](http://www.hmdb.ca/metabolites/HMDB0029666) | -- | down | 1.8305 | 0.002078 |  | - | 0.47396 | 0.57919 |
|  | 10 | 3-Cresotinic acid | C8H8O3 | 152.1473 | [HMDB0002390](http://www.hmdb.ca/metabolites/HMDB0002390) | [C14088](http://www.genome.jp/dbget-bin/www_bget?C14088) | down | 1.8113 | 0.002493 |  | - | 0.56386 | 0.50809 |
|  | 11 | Caproic acid | C6H12O2 | 116.1583 | [HMDB0000535](http://www.hmdb.ca/metabolites/HMDB0000535) | [C01585](http://www.genome.jp/dbget-bin/www_bget?C01585) | down | 1.7964 | 0.002857 |  | - | 0.26166 | 0.76068 |
|  | 12 | Indolelactic acid | C11H11NO3 | 205.2099 | [HMDB0000671](http://www.hmdb.ca/metabolites/HMDB0000671) | [C02043](http://www.genome.jp/dbget-bin/www_bget?C02043) | down | 1.7099 | 0.005827 |  | - | 0.15598 | 0.85612 |
|  | 13 | Pantothenol | C9H19NO4 | 205.2515 | [HMDB0004231](http://www.hmdb.ca/metabolites/HMDB0004231) | [C05944](http://www.genome.jp/dbget-bin/www_bget?C05944) | up | 1.6779 | 0.007373 |  | - | 1.5134 | 0.05561 |
|  | 14 | Octadecanedioic acid | C18H34O4 | 314.4602 | [HMDB0000782](http://www.hmdb.ca/metabolites/HMDB0000782) | -- | up | 1.6236 | 0.010668 |  | - | 0.6134 | 0.47068 |
|  | 15 | Creatine | C4H9N3O2 | 131.1332 | [HMDB0000064](http://www.hmdb.ca/metabolites/HMDB0000064) | [C00300](http://www.genome.jp/dbget-bin/www_bget?C00300) | up | 1.5699 | 0.014905 |  | - | 0.7121 | 0.40025 |
|  | 16 | Prostaglandin B1 | C20H32O4 | 336.4657 | [HMDB0002982](http://www.hmdb.ca/metabolites/HMDB0002982) | [C00959](http://www.genome.jp/dbget-bin/www_bget?C00959) | up | 1.4942 | 0.022815 |  | - | 0.46722 | 0.58467 |
|  | 17 | Inosine | C10H12N4O5 | 268.2261 | [HMDB0000195](http://www.hmdb.ca/metabolites/HMDB0000195) | [C00294](http://www.genome.jp/dbget-bin/www_bget?C00294) | up | 1.4859 | 0.023832 |  | - | 0.96618 | 0.24665 |
|  | 18 | Dodecanoic acid | C12H24O2 | 200.3178 | [HMDB0000638](http://www.hmdb.ca/metabolites/HMDB0000638) | [C02679](http://www.genome.jp/dbget-bin/www_bget?C02679) | down | 1.4791 | 0.024698 |  | - | 0.71324 | 0.39946 |
|  | 19 | gamma-Asarone | C12H16O3 | 208.2536 | [HMDB0029872](http://www.hmdb.ca/metabolites/HMDB0029872) | [C17821](http://www.genome.jp/dbget-bin/www_bget?C17821) | down | 1.4772 | 0.024943 |  | - | 0.18176 | 0.83262 |
|  | 20 | D-2-Hydroxyglutaric acid | C5H8O5 | 148.114 | [HMDB0000606](http://www.hmdb.ca/metabolites/HMDB0000606) | [C01087](http://www.genome.jp/dbget-bin/www_bget?C01087) | up | 1.454 | 0.028074 |  | - | 0.23692 | 0.78279 |
|  | 21 | 13-L-Hydroperoxylinoleic acid | C18H32O4 | 312.4443 | [HMDB0003871](http://www.hmdb.ca/metabolites/HMDB0003871) | [C04717](http://www.genome.jp/dbget-bin/www_bget?C04717) | up | 1.4484 | 0.028885 |  | - | 0.35607 | 0.67793 |
|  | 22 | Caprylic acid | C8H16O2 | 144.2114 | [HMDB0000482](http://www.hmdb.ca/metabolites/HMDB0000482) | [C06423](http://www.genome.jp/dbget-bin/www_bget?C06423) | down | 1.446 | 0.029221 |  | - | 0.89194 | 0.28722 |
|  | 23 | Adipic acid | C6H10O4 | 146.1412 | [HMDB0000448](http://www.hmdb.ca/metabolites/HMDB0000448) | [C06104](http://www.genome.jp/dbget-bin/www_bget?C06104) | up | 1.443 | 0.029667 |  | - | 1.1992 | 0.14289 |
|  | 24 | Melatonin | C13H16N2O2 | 232.2783 | [HMDB0001389](http://www.hmdb.ca/metabolites/HMDB0001389) | [C01598](http://www.genome.jp/dbget-bin/www_bget?C01598) | up | 1.4245 | 0.032471 |  | - | 0.61396 | 0.47026 |
|  | 25 | L-Arginine | C6H14N4O2 | 174.201 | [HMDB0000517](http://www.hmdb.ca/metabolites/HMDB0000517) | [C00062](http://www.genome.jp/dbget-bin/www_bget?C00062) | up | 1.3779 | 0.040354 |  | - | 0.57726 | 0.49784 |
|  | 26 | Phenylacetylglycine | C10H11NO3 | 193.1992 | [HMDB0000821](http://www.hmdb.ca/metabolites/HMDB0000821) | [C05598](http://www.genome.jp/dbget-bin/www_bget?C05598) | down | 1.3725 | 0.041358 |  | - | 0.32566 | 0.70427 |
|  | 27 | L-Glutamine | C5H10N2O3 | 146.1445 | [HMDB0000641](http://www.hmdb.ca/metabolites/HMDB0000641) | [C00064](http://www.genome.jp/dbget-bin/www_bget?C00064) | up | 1.3624 | 0.043263 |  | - | 0.26662 | 0.75626 |
|  | 28 | 9,10-DHOME | C18H34O4 | 314.466 | [HMDB0004704](http://www.hmdb.ca/metabolites/HMDB0004704) | [C14828](http://www.genome.jp/dbget-bin/www_bget?C14828) | up | 1.3504 | 0.045606 |  | - | 0.82633 | 0.32607 |
|  | 29 | Phenol | C6H6O | 94.1112 | [HMDB0000228](http://www.hmdb.ca/metabolites/HMDB0000228) | [C15584](http://www.genome.jp/dbget-bin/www_bget?C15584) | down | 1.3474 | 0.046215 |  | - | 0.17684 | 0.8371 |
|  | 30 | (+/-)-2-(1-Methylpropyl)-4,6-dinitrophenol | C5H10O3S | 150.196 | [HMDB32559](http://www.hmdb.ca/metabolites/HMDB32559) | [C14302](http://www.genome.jp/dbget-bin/www_bget?C14302) | up | 1.3405 | 0.047615 |  | - | 1.3502 | 0.093981 |
|  | 31 | Indole-3-methyl acetate | C11H11NO2 | 189.2105 | [HMDB0029738](http://www.hmdb.ca/metabolites/HMDB0029738) | -- | up | 1.3394 | 0.047851 |  | up | 2.3748 | 0.000227 |
|  | 32 | 4,6-Dihydroxyquinoline | C9H7NO2 | 161.1574 | [HMDB0004077](http://www.hmdb.ca/metabolites/HMDB0004077) | [C05639](http://www.genome.jp/dbget-bin/www_bget?C05639) | up | 1.3357 | 0.048604 |  | up | 2.3307 | 0.000383 |
|  | 33 | (+/-)-2-Hydroxy-4-(methylthio)butanoic acid | C5H10O3S | 150.196 | [HMDB37115](http://www.hmdb.ca/metabolites/HMDB37115) | -- | down | 1.6712 | 0.007729 |  | up | 1.6248 | 0.036873 |
|  | 34 | 9-HODE | C18H32O3 | 296.4449 | [HMDB0010223](http://www.hmdb.ca/metabolites/HMDB0010223) | -- | up | 1.4378 | 0.03043 |  | up | 1.6725 | 0.030466 |
|  | 35 | Syringic acid | C9H10O5 | 198.174 | [HMDB0002085](http://www.hmdb.ca/metabolites/HMDB0002085) | [C10833](http://www.genome.jp/dbget-bin/www_bget?C10833) | - | 1.207 | 0.080972 |  | down | 2.7295 | 8.13E-09 |
|  | 36 | 4-O-Methylgallic acid | C8H8O5 | 184.1461 | [HMDB0013198](http://www.hmdb.ca/metabolites/HMDB0013198) | -- | - | 0.33286 | 0.65468 |  | down | 2.7002 | 6.70E-08 |
|  | 37 | Miconazole | C18H14Cl4N2O | 416.129 | [HMDB0015242](http://www.hmdb.ca/metabolites/HMDB0015242) | [C08070](http://www.genome.jp/dbget-bin/www_bget?C08070) | - | 0.28782 | 0.69933 |  | down | 2.6784 | 2.15E-07 |
|  | 38 | Indoleacetic acid | C10H9NO2 | 175.184 | [HMDB0000197](http://www.hmdb.ca/metabolites/HMDB0000197) | [C00954](http://www.genome.jp/dbget-bin/www_bget?C00954) | - | 0.33303 | 0.65452 |  | up | 2.1212 | 0.002575 |
|  | 39 | N-Acetylarylamine | C8H9NO | 135.1632 | [HMDB0001250](http://www.hmdb.ca/metabolites/HMDB0001250) | [C07565](http://www.genome.jp/dbget-bin/www_bget?C07565) | - | 0.46903 | 0.52634 |  | up | 1.9329 | 0.00871 |
|  | 40 | Palmitoleic acid | C16H30O2 | 254.4082 | [HMDB0003229](http://www.hmdb.ca/metabolites/HMDB0003229) | [C08362](http://www.genome.jp/dbget-bin/www_bget?C08362) | - | 0.94995 | 0.18364 |  | up | 1.9263 | 0.009036 |
|  | 41 | Alpha-Linolenic acid | C18H30O2 | 278.4296 | [HMDB0001388](http://www.hmdb.ca/metabolites/HMDB0001388) | [C06427](http://www.genome.jp/dbget-bin/www_bget?C06427) | - | 0.83559 | 0.2474 |  | up | 1.8113 | 0.016413 |
|  | 42 | Isoquinoline | C9H7N | 129.1586 | [HMDB0034244](http://www.hmdb.ca/metabolites/HMDB0034244) | [C06323](http://www.genome.jp/dbget-bin/www_bget?C06323) | - | 0.91987 | 0.19929 |  | up | 1.778 | 0.019217 |
|  | 43 | 16-Hydroxy hexadecanoic acid | C16H32O3 | 272.4235 | [HMDB0006294](http://www.hmdb.ca/metabolites/HMDB0006294) | [C18218](http://www.genome.jp/dbget-bin/www_bget?C18218) | - | 1.1117 | 0.11273 |  | up | 1.7357 | 0.023269 |
|  | 44 | 20-Hydroxyeicosatetraenoic acid | C20H32O3 | 320.4663 | [HMDB0005998](http://www.hmdb.ca/metabolites/HMDB0005998) | [C14748](http://www.genome.jp/dbget-bin/www_bget?C14748) | - | 0.9136 | 0.20266 |  | up | 1.702 | 0.026928 |
|  | 45 | L-Lactic acid | C3H6O3 | 90.0779 | [HMDB0000190](http://www.hmdb.ca/metabolites/HMDB0000190) | [C00186](http://www.genome.jp/dbget-bin/www_bget?C00186) | - | 0.64973 | 0.37529 |  | up | 1.6837 | 0.029085 |
|  | 46 | 3-Indolebutyric acid | C12H13NO2 | 203.2371 | [HMDB0002096](http://www.hmdb.ca/metabolites/HMDB0002096) | [C11284](http://www.genome.jp/dbget-bin/www_bget?C11284) | - | 0.94398 | 0.18668 |  | up | 1.6744 | 0.030227 |
|  | 47 | Metronidazole | C6H9N3O3 | 171.154 | [HMDB0015052](http://www.hmdb.ca/metabolites/HMDB0015052) | [C07203](http://www.genome.jp/dbget-bin/www_bget?C07203) | - | 1.27 | 0.06371 |  | down | 1.6167 | 0.038051 |
|  | 48 | Linoleic acid | C18H32O2 | 280.4455 | [HMDB0000673](http://www.hmdb.ca/metabolites/HMDB0000673) | [C01595](http://www.genome.jp/dbget-bin/www_bget?C01595) | - | 0.27515 | 0.71206 |  | down | 1.5612 | 0.046882 |
| **AT7** |  |  |  |  |  |  |  |  |  |  |  |  |  |
|  | 1 | Phthalic acid | C8H6O4 | 166.1308 | [HMDB0002107](http://www.hmdb.ca/metabolites/HMDB0002107) | [C01606](http://www.genome.jp/dbget-bin/www_bget?C01606) | down | 2.3683 | 1.11E-08 |  | - | 0.61033 | 0.46209 |
|  | 2 | Dibutyl phthalate | C16H22O4 | 278.3435 | [HMDB0033244](http://www.hmdb.ca/metabolites/HMDB0033244) | [C14214](http://www.genome.jp/dbget-bin/www_bget?C14214) | down | 2.3676 | 1.22E-08 |  | - | 0.51433 | 0.53699 |
|  | 3 | 4-Coumaryl alcohol | C9H10O2 | 150.1745 | [HMDB0003654](http://www.hmdb.ca/metabolites/HMDB0003654) | [C02646](http://www.genome.jp/dbget-bin/www_bget?C02646) | down | 2.3613 | 2.44E-08 |  | - | 0.12767 | 0.87923 |
|  | 4 | Benzoic acid | C7H6O2 | 122.123 | [HMDB0001870](http://www.hmdb.ca/metabolites/HMDB0001870) | [C00539](http://www.genome.jp/dbget-bin/www_bget?C00539) | down | 2.2892 | 2.25E-06 |  | - | 0.4447 | 0.59434 |
|  | 5 | Prostaglandin F2a | C20H34O5 | 354.481 | [HMDB0001139](http://www.hmdb.ca/metabolites/HMDB0001139) | [C00639](http://www.genome.jp/dbget-bin/www_bget?C00639) | up | 1.9652 | 0.00122 |  | - | 0.23712 | 0.7775 |
|  | 6 | Uric acid | C5H4N4O3 | 168.1103 | [HMDB0000289](http://www.hmdb.ca/metabolites/HMDB0000289) | [C00366](http://www.genome.jp/dbget-bin/www_bget?C00366) | up | 1.9451 | 0.0015 |  | - | 0.097052 | 0.90807 |
|  | 7 | Isoquinoline | C9H7N | 129.1586 | [HMDB0034244](http://www.hmdb.ca/metabolites/HMDB0034244) | [C06323](http://www.genome.jp/dbget-bin/www_bget?C06323) | up | 1.7535 | 0.007295 |  | - | 1.1971 | 0.13324 |
|  | 8 | Indoleacetic acid | C10H9NO2 | 175.184 | [HMDB0000197](http://www.hmdb.ca/metabolites/HMDB0000197) | [C00954](http://www.genome.jp/dbget-bin/www_bget?C00954) | down | 1.7107 | 0.009683 |  | - | 0.36859 | 0.6596 |
|  | 9 | 2,4-Dihydroxybenzoic acid | C7H6O4 | 154.121 | [HMDB0029666](http://www.hmdb.ca/metabolites/HMDB0029666) | -- | down | 1.6324 | 0.015494 |  | - | 0.28557 | 0.73339 |
|  | 10 | Taurochenodesoxycholic acid | C26H45NO6S | 499.704 | [HMDB0000951](http://www.hmdb.ca/metabolites/HMDB0000951) | [C05465](http://www.genome.jp/dbget-bin/www_bget?C05465) | down | 1.6139 | 0.017177 |  | - | 1.1159 | 0.1648 |
|  | 11 | Spermidine | C7H19N3 | 145.2459 | [HMDB0001257](http://www.hmdb.ca/metabolites/HMDB0001257) | [C00315](http://www.genome.jp/dbget-bin/www_bget?C00315) | up | 1.6007 | 0.018453 |  | - | 0.22506 | 0.78857 |
|  | 12 | Capric acid | C10H20O2 | 172.2646 | [HMDB0000511](http://www.hmdb.ca/metabolites/HMDB0000511) | [C01571](http://www.genome.jp/dbget-bin/www_bget?C01571) | down | 1.5737 | 0.021281 |  | - | 0.51876 | 0.53342 |
|  | 13 | 3-Indolebutyric acid | C12H13NO2 | 203.2371 | [HMDB0002096](http://www.hmdb.ca/metabolites/HMDB0002096) | [C11284](http://www.genome.jp/dbget-bin/www_bget?C11284) | up | 1.5492 | 0.024123 |  | - | 1.2597 | 0.11185 |
|  | 14 | trans-Aconitic acid | C6H6O6 | 174.1082 | [HMDB0000958](http://www.hmdb.ca/metabolites/HMDB0000958) | [C02341](http://www.genome.jp/dbget-bin/www_bget?C02341) | up | 1.5344 | 0.025958 |  | - | 0.20893 | 0.80345 |
|  | 15 | Phenol | C6H6O | 94.1112 | [HMDB0000228](http://www.hmdb.ca/metabolites/HMDB0000228) | [C15584](http://www.genome.jp/dbget-bin/www_bget?C15584) | down | 1.5252 | 0.027144 |  | - | 0.33388 | 0.69015 |
|  | 16 | N-Acetylornithine | C7H14N2O3 | 174.1977 | [HMDB0003357](http://www.hmdb.ca/metabolites/HMDB0003357) | [C00437](http://www.genome.jp/dbget-bin/www_bget?C00437) | down | 1.5208 | 0.027735 |  | - | 0.21777 | 0.79529 |
|  | 17 | Glutaconic acid | C5H6O4 | 130.0987 | [HMDB0000620](http://www.hmdb.ca/metabolites/HMDB0000620) | [C02214](http://www.genome.jp/dbget-bin/www_bget?C02214) | up | 1.45 | 0.03843 |  | - | 0.73975 | 0.36976 |
|  | 18 | (+/-)-2-(1-Methylpropyl)-4,6-dinitrophenol | C10H12N2O5 | 240.2127 | [HMDB32559](http://www.hmdb.ca/metabolites/HMDB32559) | [C14302](http://www.genome.jp/dbget-bin/www_bget?C14302) | up | 1.4308 | 0.041777 |  | - | 1.2863 | 0.10351 |
|  | 19 | Dodecanoic acid | C12H24O2 | 200.3178 | [HMDB0000638](http://www.hmdb.ca/metabolites/HMDB0000638) | [C02679](http://www.genome.jp/dbget-bin/www_bget?C02679) | down | 1.4024 | 0.047086 |  | - | 0.61428 | 0.45913 |
|  | 20 | Lenticin | C14H18N2O2 | 246.3049 | [HMDB0061115](http://www.hmdb.ca/metabolites/HMDB0061115) | [C09213](http://www.genome.jp/dbget-bin/www_bget?C09213) | down | 2.1115 | 0.000184 |  | up | 1.5232 | 0.047123 |
|  | 21 | Biotin | C10H16N2O3S | 244.311 | [HMDB0000030](http://www.hmdb.ca/metabolites/HMDB0000030) | [C00120](http://www.genome.jp/dbget-bin/www_bget?C00120) | up | 1.9662 | 0.001208 |  | down | 1.6704 | 0.02595 |
|  | 22 | Pyruvic acid | C3H4O3 | 88.0621 | [HMDB0000243](http://www.hmdb.ca/metabolites/HMDB0000243) | [C00022](http://www.genome.jp/dbget-bin/www_bget?C00022) | up | 1.9389 | 0.001596 |  | up | 1.8879 | 0.008686 |
|  | 23 | Miconazole | C18H14Cl4N2O | 416.129 | [HMDB0015242](http://www.hmdb.ca/metabolites/HMDB0015242) | [C08070](http://www.genome.jp/dbget-bin/www_bget?C08070) | up | 1.9098 | 0.002108 |  | down | 2.6764 | 2.68E-09 |
|  | 24 | Phytosphingosine | C18H39NO3 | 317.5072 | [HMDB0004610](http://www.hmdb.ca/metabolites/HMDB0004610) | [C12144](http://www.genome.jp/dbget-bin/www_bget?C12144) | up | 1.7227 | 0.008966 |  | down | 1.5821 | 0.037552 |
|  | 25 | Cyclohexylamine | C6H13N | 99.1741 | [HMDB0031404](http://www.hmdb.ca/metabolites/HMDB0031404) | [C00571](http://www.genome.jp/dbget-bin/www_bget?C00571) | down | 1.4866 | 0.032588 |  | down | 2.6929 | 2.90E-10 |
|  | 26 | N-Acetylarylamine | C8H9NO | 135.1632 | [HMDB0001250](http://www.hmdb.ca/metabolites/HMDB0001250) | [C07565](http://www.genome.jp/dbget-bin/www_bget?C07565) | - | 0.27944 | 0.71965 |  | down | 2.6879 | 6.32E-10 |
|  | 27 | 4-O-Methylgallic acid | C8H8O5 | 184.1461 | [HMDB0013198](http://www.hmdb.ca/metabolites/HMDB0013198) | -- | - | 0.91204 | 0.22501 |  | down | 2.6645 | 8.76E-09 |
|  | 28 | Syringic acid | C9H10O5 | 198.174 | [HMDB0002085](http://www.hmdb.ca/metabolites/HMDB0002085) | [C10833](http://www.genome.jp/dbget-bin/www_bget?C10833) | - | 0.23745 | 0.76059 |  | down | 2.6049 | 3.53E-07 |
|  | 29 | 4-Nitrophenol | C6H5NO3 | 139.1088 | [HMDB0001232](http://www.hmdb.ca/metabolites/HMDB0001232) | [C00870](http://www.genome.jp/dbget-bin/www_bget?C00870) | - | 0.40512 | 0.60143 |  | down | 2.095 | 0.002142 |
|  | 30 | Hypoxanthine | C5H4N4O | 136.1115 | [HMDB0000157](http://www.hmdb.ca/metabolites/HMDB0000157) | [C00262](http://www.genome.jp/dbget-bin/www_bget?C00262) | - | 0.15897 | 0.83857 |  | up | 1.8716 | 0.009537 |
|  | 31 | Taurine | C2H7NO3S | 125.147 | [HMDB0000251](http://www.hmdb.ca/metabolites/HMDB0000251) | [C00245](http://www.genome.jp/dbget-bin/www_bget?C00245) | - | 0.20917 | 0.7885 |  | up | 1.7836 | 0.015242 |
|  | 32 | Adipic acid | C6H10O4 | 146.1412 | [HMDB0000448](http://www.hmdb.ca/metabolites/HMDB0000448) | [C06104](http://www.genome.jp/dbget-bin/www_bget?C06104) | - | 0.50581 | 0.51257 |  | down | 1.7831 | 0.01528 |
|  | 33 | L-Lactic acid | C3H6O3 | 90.0779 | [HMDB0000190](http://www.hmdb.ca/metabolites/HMDB0000190) | [C00186](http://www.genome.jp/dbget-bin/www_bget?C00186) | - | 0.77931 | 0.30512 |  | up | 1.7236 | 0.020394 |
|  | 34 | 5-KETE | C20H30O3 | 318.4504 | [HMDB0010217](http://www.hmdb.ca/metabolites/HMDB0010217) | [C14732](http://www.genome.jp/dbget-bin/www_bget?C14732) | - | 0.39334 | 0.6122 |  | down | 1.6646 | 0.026623 |
|  | 35 | Alpha-Linolenic acid | C18H30O2 | 278.4296 | [HMDB0001388](http://www.hmdb.ca/metabolites/HMDB0001388) | [C06427](http://www.genome.jp/dbget-bin/www_bget?C06427) | - | 0.41756 | 0.59015 |  | up | 1.6382 | 0.029817 |
|  | 36 | Glyceraldehyde | C3H6O3 | 90.0779 | [HMDB0001051](http://www.hmdb.ca/metabolites/HMDB0001051) | [C02154](http://www.genome.jp/dbget-bin/www_bget?C02154) | - | 0.87358 | 0.24677 |  | up | 1.5764 | 0.038401 |

**TABLE S7 |** The predominant potential serum biomarkers based on the significant metabolic pathways

| **Stage** | **Metabolism** | **Pathway name (*P* value)** | **Match metabolites** |
| --- | --- | --- | --- |
| **BT1**  **（CON vs. TS+GA）** | Amino acid metabolism | Lysine degradation (0.0096529) | L-Lysine; L-Carnitine |
|  | Lipid metabolism | Sphingolipid metabolism (0.014829) | Phytosphingosine |
|  | Amino acid metabolism | Tyrosine metabolism (0.018281) | Homovanillic acid; Pyruvic acid |
|  | Carbohydrate metabolism | Glycolysis / Gluconeogenesis (0.033673) | Pyruvic acid; L-Lactic acid |
|  | Carbohydrate metabolism | Pyruvate metabolism (0.033673) | Pyruvic acid; L-Lactic acid |
| **BT1**  **（TS vs. TS+GA）** | Lipid metabolism | Sphingolipid metabolism (0.0021967) | Phytosphingosine |
|  | Amino acid metabolism | Lysine degradation (0.0027051) | L-Lysine; L-Carnitine |
|  | Metabolism of other amino acid | Taurine and hypotaurine metabolism (0.032098) | Taurine |
|  | Metabolism of other amino acid | Glutathione metabolism (0.046417) | L-Glutamic acid; Pyroglutamic acid; Spermidine |
| **AT1**  **（CON vs. TS）** | Amino acid metabolism | Glycine, serine and threonine metabolism (0.013893) | Creatine; Pyruvic acid |
|  | Amino acid metabolism | Arginine and proline metabolism (0.014041) | L-Arginine; Creatine; Spermidine; 4-Hydroxyproline; L-Proline; L-Glutamic acid; Pyruvic acid |
|  | Amino acid metabolism | Arginine biosynthesis (0.028949) | L-Glutamic acid; L-Arginine; N-Acetylornithine; Citrulline; L-Glutamine |
|  | Carbohydrate metabolism | Glyoxylate and dicarboxylate metabolism (0.029156) | Citric acid; L-Glutamic acid; Pyruvic acid; L-Glutamine |
|  | Amino acid metabolism | Alanine, aspartate and glutamate metabolism (0.029156) | L-Glutamic acid; L-Glutamine; Citric acid; Pyruvic acid |
|  | Nucleotide metabolism | Pyrimidine metabolism (0.047306) | L-Glutamine; Ureidopropionic acid |
|  | Metabolism of other amino acid | D-Glutamine and D-glutamate metabolism (0.048425) | L-Glutamic acid; L-Glutamine |
|  | Energy metabolism | Nitrogen metabolism (0.048425) | L-Glutamic acid; L-Glutamine |
| **AT1**  **（CON vs. TS+GA）** | Lipid metabolism | Alpha-Linolenic acid metabolism (0.0021409) | Alpha-Linolenic acid |
|  | Carbohydrate metabolism | Glycolysis / Gluconeogenesis (0.018272) | Pyruvic acid; L-Lactic acid |
|  | Carbohydrate metabolism | Pyruvate metabolism (0.018272) | Pyruvic acid; L-Lactic acid |
|  | Lipid metabolism | Biosynthesis of unsaturated fatty acids (0.027752) | Oleic acid; Linoleic acid; Arachidonic acid; Alpha-Linolenic acid |
|  | Lipid metabolism | Linoleic acid metabolism (0.029407) | Linoleic acid; 13-L-Hydroperoxylinoleic acid |
| **AT1**  **（TS vs. TS+GA）** | Carbohydrate metabolism | Glycolysis / Gluconeogenesis (0.024938) | Pyruvic acid; L-Lactic acid |
|  | Carbohydrate metabolism | Pyruvate metabolism (0.024938) | Pyruvic acid; L-Lactic acid |
|  | Lipid metabolism | Alpha-Linolenic acid metabolism (0.028704) | Alpha-Linolenic acid |
|  | Lipid metabolism | Biosynthesis of unsaturated fatty acids (0.039808) | Oleic acid; Linoleic acid; Arachidonic acid; Alpha-Linolenic acid |
|  | Lipid metabolism | Linoleic acid metabolism (0.046794) | Linoleic acid; 13-L-Hydroperoxylinoleic acid |
| **AT7**  **（CON vs. TS）** | Amino acid metabolism | Tyrosine metabolism (0.0015465) | Homovanillic acid; Pyruvic acid |
|  | Amino acid metabolism | Cysteine and methionine metabolism (0.0021331) | Pyruvic acid |
|  | Lipid metabolism | Sphingolipid metabolism (0.0098736) | Phytosphingosine |
|  | Lipid metabolism | Fatty acid biosynthesis (0.029089) | Dodecanoic acid; Capric acid |
| **AT7**  **（CON vs. TS+GA）** | Amino acid metabolism | Cysteine and methionine metabolism (5.8268E-5) | Pyruvic acid |
|  | Amino acid metabolism | Tyrosine metabolism (7.4722E-5) | Homovanillic acid; Pyruvic acid |
|  | Carbohydrate metabolism | Glycolysis / Gluconeogenesis (0.0048281) | Pyruvic acid; L-Lactic acid |
|  | Carbohydrate metabolism | Pyruvate metabolism (0.0048281) | Pyruvic acid; L-Lactic acid |
|  | Carbohydrate metabolism | Fructose and mannose metabolism (0.00765) | Glyceraldehyde |
|  | Lipid metabolism | Glycerolipid metabolism (0.00765) | Glyceraldehyde |
|  | Nucleotide metabolism | Purine metabolism (0.01166) | L-Glutamine; Hypoxanthine; Inosine; Uric acid |
|  | Amino acid metabolism | Valine, leucine and isoleucine degradation (0.029608) | L-Valine; L-Isoleucine |
|  | Amino acid metabolism | Valine, leucine and isoleucine biosynthesis (0.029608) | L-Valine; L-Isoleucine |
|  | Amino acid metabolism | Lysine degradation (0.040328) | L-Lysine; Carnitine |
|  | Lipid metabolism | Fatty acid biosynthesis (0.049822) | Dodecanoic acid; Capric acid |
| **AT7**  **（TS vs. TS+GA）** | Amino acid metabolism | Cysteine and methionine metabolism (0.0093482) | Pyruvic acid |
|  | Carbohydrate metabolism | Glycolysis / Gluconeogenesis (0.01599) | Pyruvic acid; L-Lactic acid |
|  | Carbohydrate metabolism | Pyruvate metabolism (0.01599) | Pyruvic acid; L-Lactic acid |
|  | Nucledtide metabolism | Purine metabolism (0.017779) | L-Glutamine; Hypoxanthine; Inosine; Uric acid |
|  | Lipid metabolism | Primary bile acid biosynthesis (0.021779) | Chenodeoxycholic acid; Taurine; Cholic acid; Taurochenodesoxycholic acid |
|  | Metabolism of other amino acid | Taurine and hypotaurine metabolism (0.023085) | Taurine |
|  | Lipid metabolism | Alpha-Linolenic acid metabolism (0.025954) | Alpha-Linolenic acid |
|  | Carbohydrate metabolism | Fructose and mannose metabolism (0.03322) | Glyceraldehyde |
|  | Lipid metabolism | Glycerolipid metabolism (0.03322) | Glyceraldehyde |
